# Cold exposure stimulates cross-tissue metabolic rewiring to fuel glucose-dependent thermogenesis in brown adipose tissue

**DOI:** 10.1126/sciadv.adt7369

**Published:** 2025-06-11

**Authors:** Harry B. Cutler, Sigrid Jall-Rogg, Senthil Thillainadesan, Kristen C. Cooke, Stewart W. C. Masson, James M. Sligar, Jonathan G. Crowston, Luke Carroll, Jacqueline Stöckli, David E. James, Søren Madsen

**Affiliations:** ^1^ School of Life and Environmental Sciences, University of Sydney, Camperdown, New South Wales, Australia.; ^2^ Charles Perkins Centre, University of Sydney, Camperdown, New South Wales, Australia.; ^3^ Save Sight Institute, Faculty of Medicine, University of Sydney, Camperdown, New South Wales, Australia.; ^4^ School of Medical Sciences, University of Sydney, Camperdown, New South Wales, Australia.

## Abstract

To gain insight into the root causes of metabolic dysfunction, it is essential to understand how tissues communicate and coordinate their metabolic functions. Here, we sought to address this in the context of cold exposure, a well-studied metabolic perturbation. We performed proteomics across six metabolic tissues and plasma, quantifying 11,394 proteins. Beginning our investigation in brown adipose tissue (BAT), we identified a mechanism to explain enhanced glucose utilization in cold-adapted BAT. This was characterized by select remodeling of upper glycolysis and pentose cycling to increase oxygen consumption, likely by increasing uncoupling protein 1 activity through the production of reactive oxygen species. Cold-induced remodeling of the plasma proteome appeared to underpin the ability of BAT to modify its fuel preference, stimulating lipolysis in white adipose tissue and glucose production in the liver. These findings emphasize the importance of considering metabolic adaptations in the context of the whole body and suggest overlap between the mechanisms of cold adaptation and obesity.

## Teaser

UCP1 is activated by pentose cycling in brown adipose tissue, leading to systemic metabolic adaptation.

## INTRODUCTION

Cold exposure has profound effects on metabolism, including increased energy expenditure (*1*), enhanced lipolysis (*2*, *3*) and improved insulin sensitivity (*4*, *5*). In the context of growing pressure placed on health care systems worldwide by obesity and related metabolic complications (*6*), targeting these adaptations is therapeutically attractive. Given the strong inverse relationship between brown adipose tissue (BAT) activity and obesity in humans (*7*), many studies have sought to understand the mechanisms by which BAT contributes to systemic metabolism (*8*). While there can be no debate that BAT plays a central role in adaptive thermogenesis, the potential for other organs to contribute to this process should not be neglected. For example, enhanced glucose consumption in cold-adapted BAT (*9*) is paralleled by an increase in hepatic gluconeogenesis and glycogenolysis (*10*, *11*), suggesting a critical role of the liver in supplying metabolic substrates to BAT. Fatty acids (FAs) are also thought to be a key source of energy for cold-induced BAT, and basal lipolysis in white adipose tissue is likely up-regulated to meet this demand. It appears, therefore, that coordinated metabolic adaptations across many tissues are required to enable BAT to fulfill its function.

While great advances have been made in developing strategies to activate human BAT (*12*–*14*) and stimulate thermogenic adipogenesis (*15*, *16*), the extent to which the whole-body response to cold is orchestrated by tissues other than BAT is not well understood. The need for metabolic coordination between organs seems to be actively communicated during cold adaptation, evidenced by the release of temperature-regulated secretory factors into the circulation, which have been shown to modulate metabolism in both peripheral tissues and BAT itself (*17*, *18*). These observations raise the possibility that adaptations in one tissue might stimulate downstream adaptation in others. To fully grasp the impact of cold-induced changes in an individual tissue, therefore, it is necessary to assess changes in the broader context of the whole body.

Consistent with cold exposure necessitating distinct adaptations across multiple organs, a recent study examining the transcriptional response to cold exposure found that changes were highly tissue specific and that a unifying signature could not be identified across tissues (*19*). Because proteins provide a crucial link between the sensing of environmental perturbations and the physiological adaptations that follow, here, we leveraged a proteomic approach to study the functional effects of cold exposure. Analyses were performed in visceral epididymal white adipose tissues (eWAT) and subcutaneous white adipose tissues (sWAT), BAT, quadriceps muscle, liver, hypothalamus, and plasma from mice housed under thermoneutral (30°C) or cold (5°C) conditions for 3 weeks. To encourage community engagement with these data where phenotypes of interest are affected by cold exposure, all data have been made publicly available via an interactive web tool (https://bigproteomics.shinyapps.io/ColdAdaptation/). This tool also facilitates a direct download of processed proteomic data.

Comprehensive analysis of how tissues compensate for increased metabolic demands upon cold exposure indicated the existence of a cold-induced metabolic cross-talk involving white adipose tissue, liver, and BAT that ultimately functions to increase glucose supply to BAT. Regulated changes to the plasma proteome are likely required to maintain this pathway, exemplified by cold-induced secretion of fetuin A from the liver, which has previously been reported to enhance lipolysis in adipose tissues. Last, and to emphasize the broader utility of this multitissue approach, we interrogated protein changes in cold-exposed BAT against those in diet-induced obese mice. Changes overlapped inversely between perturbations such that proteins that were up-regulated by cold exposure were down-regulated in diet-induced mice. These data support the hypothesis that impaired BAT function may contribute to obesity and implicate altered mRNA processing as a potential driver of impaired BAT function.

## RESULTS

### Multitissue proteomics of cold exposure

The overarching goal of this study was to understand the whole-body response to cold. Many studies focus on single cell types such as brown or beige adipocytes, even though others such as myocytes and hepatocytes likely also play important roles. To explore this, we examined key biological processes involved in cold adaptation through multitissue proteomic analysis. The advantage of this approach is that it is completely unbiased and, provided sufficient depth of analysis, has the potential to provide mechanistic insight into complex physiological processes and systems-level changes.

**Fig. 1.**
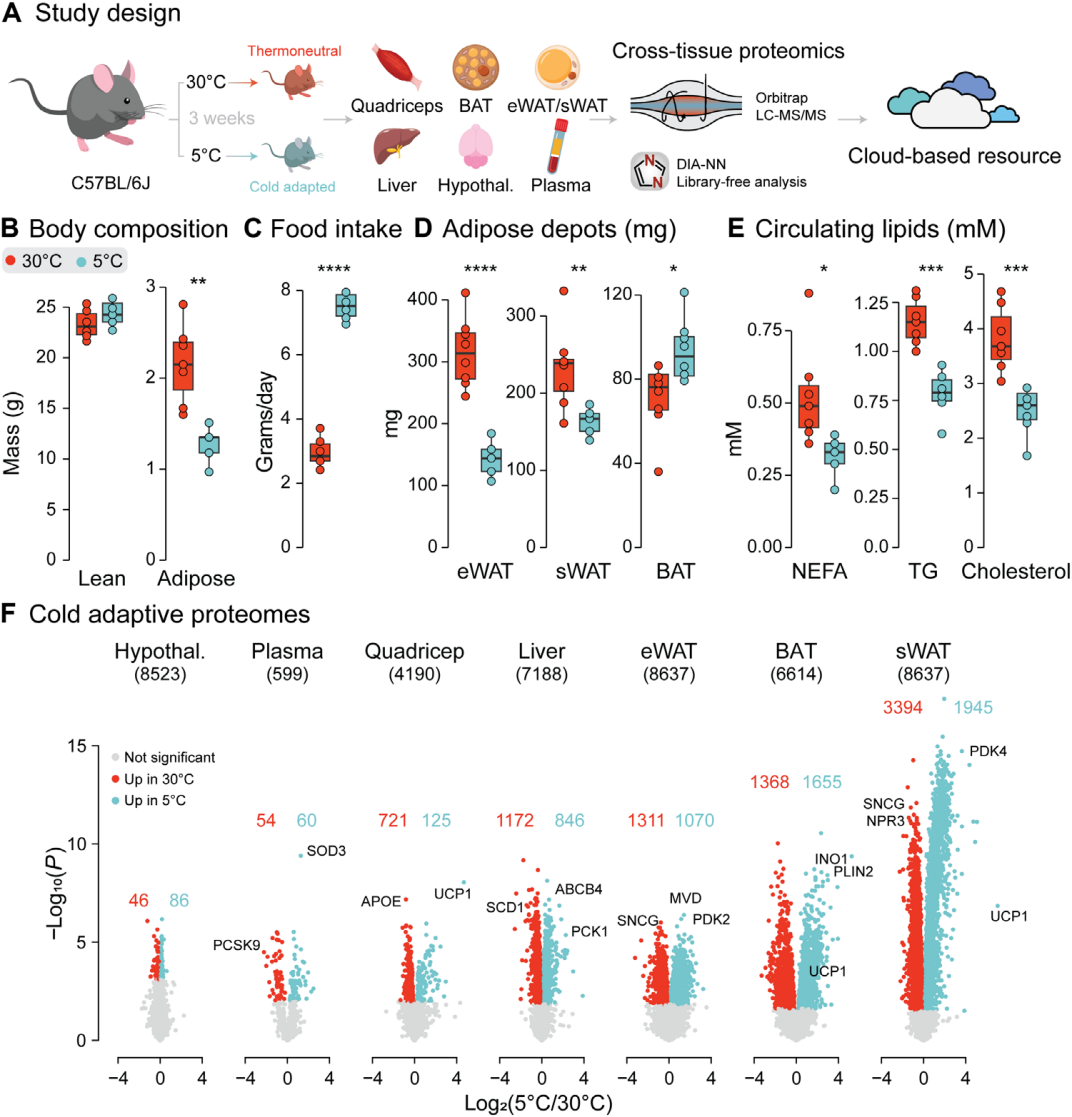
Multitissue proteomics of cold exposure. (**A**) Overview of experimental design. WAT, white adipose tissue; Hypothal, hypothalamus. LC-MS/MS, liquid chromatography–tandem mass spectrometry. (**B**) Mass of lean and adipose tissues in mice housed at either 30°C (red) or 5°C (blue) for 3 weeks, determined by EchoMRI. (**C**) Food intake during the final week of cold adaptation. (**D**) Adipose depot weights. (**E**) Concentrations of lipid species in plasma. (**F**) Volcano plots comparing proteomes of mice housed at either 5° or 30°C. Tissues are ordered by the proportion of the proteome significantly regulated by cold adaptation. Total protein identifications are reported in brackets, as well as the number of significantly regulated proteins for each tissue. Significance was determined by a two-tailed Student’s *t* test accounting for multiple testing using an FDR correction. Significance is represented with *P < 0.05, **P < 0.01, ***P < 0.001, and ****P < 0.0001. *n* = 5 to 8 per condition.

To stimulate systemic thermal adaptation, mice were housed under thermoneutral (30°C) or cold (5°C) conditions for 3 weeks (Fig. 1A). Cold exposure resulted in a twofold increase in food consumption but decreased adiposity (Fig. 1, B and C), consistent with increased fuel utilization to maintain euthermia. White adipose tissues, including sWAT and eWAT, decreased in size after sustained cold exposure, as widely reported by others (*20*). BAT mass was significantly increased (Fig. 1D), consistent with proliferation of brown adipose precursor cells (*21*, *22*). Circulating lipid species were decreased (Fig. 1E).

To characterize the molecular changes responsible for coordinating energy homeostasis in cold-adapted mice, we profiled plasma and an array of metabolically active tissues including BAT, eWAT, sWAT, quadriceps muscle, liver, and hypothalamus by label-free quantitative proteomics. Overall, 11,394 unique proteins were identified across tissues, serving as a comprehensive resource of cold adaptation at the molecular level (Fig. 1F). The extent of proteomic remodeling varied widely between tissues, ranging from 15% in hypothalamus to 62% in sWAT, consistent with the hypothesis that metabolic rewiring in many tissues is required to orchestrate the whole-body response to cold. Unexpectedly, cold-induced remodeling occurred to a similar extent in the liver and eWAT. Below, we provide a summary of key changes in the proteomes of these tissues, from which we infer potential functional changes. In many cases, such as increased glucose utilization in BAT, inferences are supported by external physiological data, reinforcing the validity of the study approach. In cases where physiological studies are yet to be performed, we recognize the need for further functional validation.

The canonical marker of adaptive thermogenesis, uncoupling protein 1 (UCP1), was significantly up-regulated upon cold adaptation in all tissues where it was detected (BAT, sWAT, and quadriceps). The overall levels of UCP1 in muscle were much lower than in other tissues, and so, this finding likely indicates contamination of the muscle tissue proteome by adipocytes (*23*). Minor changes were observed in the hypothalamus, perhaps suggesting that cold adaptation primarily affects metabolic tissues. Consistent with the known function of cold exposure in promoting fatty acid utilization by many tissues, PDK4 (pyruvate dehydrogenase kinase 4), a master regulator of glucose-to-lipid fuel switching, was up-regulated 1.8-, 4.1-, and 12.5-fold in quadriceps, eWAT, and sWAT, respectively. In line with enhanced lipid release into the circulation upon cold exposure (*2*, *3*), the levels of NPR3 (natriuretic peptide receptor 3), an inhibitor of lipolysis (*24*), were reduced by 80 to 90% in eWAT and sWAT. Together, these examples support the use of proteomics as a tool to study physiological adaptations to cold exposure across tissues, and future studies should seek to use this dataset where phenotypes of interest are affected by cold adaptation.

### BAT thermogenesis is regulated by substrate switching

**Fig. 2.**
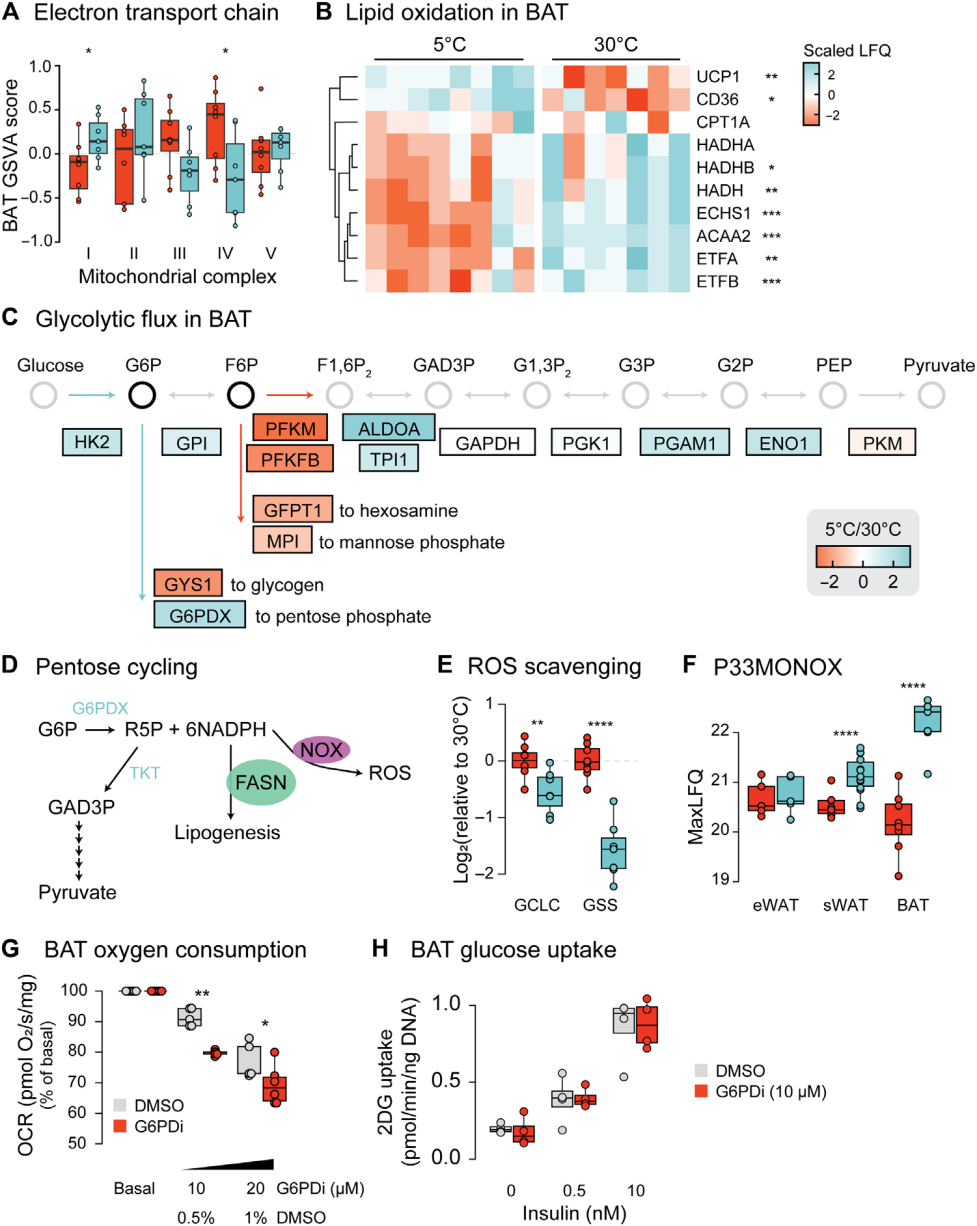
BAT thermogenesis is regulated by substrate switching. (**A**) Results of gene set variation analysis comparing the levels of the mitochondrial ETC in BAT. (**B**) Heatmap showing scaled abundance for proteins involved in fatty acid metabolism for all samples. LFQ, label-free quantification. (**C**) Schematic of cold-induced changes in BAT glycolysis and related metabolic pathways. Colors indicate cold-induced changes. Metabolite names are abbreviated as follows: G6P, glucose-6-phosphate; F6P, fructose-6-phosphate; F1,6P_2_, fructose-1,6-bisphosphate; GAD3P, glyceraldehyde-3-phosphate; G1,3P_2_, glycerate-1,3-bisphospate; G3P, glycerate-3-phosphate; G2P, glycerate-2-phosphate; PEP, phosphoenolpyruvate; GAPDH, glyceraldehyde-3-phosphate dehydrogenase. (**D**) Schematic of metabolites and key proteins involved in pentose cycling. (**E**) Cold-induced changes in protein abundance in BAT for proteins involved in glutathione synthesis, normalized to the thermoneutral mean. (**F**) Abundance of P33MONOX across adipose tissues. (**G**) BAT oxygen consumption rate (OCR) measured by Oroboros with or without G6PDi. (**H**) Insulin-stimulated ex vivo glucose uptake into BAT measured using ^3^H-2-deoxy-glucose, normalized to DNA content of each sample. Significance was determined using a two-tailed Student’s *t* test. Significance is represented with a *P < 0.05, **P < 0.01, ***P < 0.001, and ****P < 0.0001. *n* = 5 to 8 per condition.

BAT is the primary tissue responsible for nonshivering thermogenesis during cold (*8*) and undergoes substantial proteomic remodeling after cold exposure (Fig. 1F). While it is generally believed that fatty acids are a primary fuel source for BAT (*25*–*27*), more recent evidence suggests that glucose is also a critical fuel source during cold adaptation. For example, Panic *et al.* (*28*) demonstrated that deletion of the mitochondrial pyruvate carrier in BAT markedly impairs cold tolerance, indicating that glucose-derived pyruvate plays a key role in thermogenesis. To gain insight into the fuel preferences of cold-adapted BAT, we performed a targeted analysis of the effects of cold on proteins of the electron transport chain (ETC) in BAT (Fig. 2A). This demonstrated a significant increase in the abundance of 22 subunits of complex 1. In comparison to complex 2, which accepts electrons from fatty acid oxidation, complex 1 primarily facilitates the entry of carbohydrate-derived electrons into the ETC. Thus, an increase in the complex 1–to–complex 2 ratio is consistent with enhanced carbohydrate oxidation. Conversely, we also observed significant decreases in the levels of many proteins involved in fatty acid oxidation (Fig. 2B), including enzymes involved in short chain fatty acid oxidation (HADH, ECHS1, and ACAA2) and proteins that carry electrons from fatty acid breakdown into the ETC (ETFA and ETFB).

Although labeled carbon tracing studies have identified enhanced glucose utilization in cold-adapted BAT (*9*, *10*), the mechanism for this effect is not known. Here, we observed that many glycolytic proteins were significantly up-regulated in BAT by cold exposure, providing a potential mechanistic explanation for these metabolic adaptations. However, the levels of phosphofructokinase, which catalyzes the rate-limiting step of proximal glycolysis, were significantly down-regulated by cold (Fig. 2C). This is inconsistent with an overall increase in the flux of glucose via the entire glycolytic pathway. Instead, these data suggest a model where cold exposure might lead to accumulation of fructose-6-phosphate and/or glucose-6-phosphate because these are readily interconverted by glucose-6-phosphate isomerase. Of the known metabolic pathways involving these metabolites, only the pentose phosphate pathway (PPP) contained proteins up-regulated by cold, most notably glucose-6-phosphate dehydrogenase (G6PD; G6PD X-linked) which catalyzes the rate-limiting step for glucose entry into the PPP. We first hypothesized that up-regulation of glucose flux into the PPP might provide a mechanism for enhanced proliferation for cold-exposed brown adipocytes by providing increased nucleotide biosynthetic intermediates (*21*, *22*). However, the abundance of ribose-phosphate diphosphokinase (PRPS1), the rate-limiting step of nucleotide synthesis from ribose-5-P (R5P), was down-regulated fourfold. Thus, we wondered whether carbon shuttling into the PPP might serve an alternative purpose in mature brown adipocytes.

Enhanced flow of carbons into the PPP has previously been demonstrated in neutrophils, where the “pentose cycle” is integral to the ability of neutrophils to undergo activation (*29*). The pentose cycle involves flux of carbon through PPP to R5P, which then reenters glycolysis at the level of glyceraldehyde-3-phosphate (GAD3P; Fig. 2D), having sacrificed one pyruvate for six NADPH. Consistent with enhanced pentose cycling in cold-adapted BAT, the proteins that return R5P to lower glycolysis, the transketolases (TKT and TKTL2), were 1.9- and 1.5-fold up-regulated by cold exposure. In neutrophils, this reconfiguration fuels superoxide production via NADPH (reduced form of nicotinamide adenine dinucleotide phosphate) oxidases (NOXs) and so we considered whether elevated reactive oxygen species might serve an inherent function in cold-adapted BAT. Increased superoxide production has previously been demonstrated to enhance UCP1 activity (*30*, *31*), raising the possibility that a shift toward pentose cycling may serve to enhance the thermogenic capacity of BAT. Although it is also possible that enhanced NADPH synthesis might fuel lipolysis, this seems unlikely as we did not observe any changes to proteins involved in de novo lipogenesis (DNL).

Consistent with a programmed increase in cytoplasmic superoxide levels, levels of glutamate-cysteine ligase and glutathione synthase, both rate-limiting enzymes in glutathione synthesis, were strongly down-regulated by cold adaptation (Fig. 2E). Echtay *et al.* (*30*) showed that superoxide generated outside the mitochondria is sufficient to promote UCP1 activation, and so, there is no requirement for UCP1 to be activated solely by mitochondrial superoxide.

The pentose cycle generates superoxide through the action of NOXs. While we failed to detect any well-characterized NOXs in our proteomic dataset, suggesting that they are either of low abundance or not expressed in brown adipocytes, we did detect abundant expression of a less well-characterized NOX, P33MONOX. This protein was up-regulated fourfold in BAT from cold-exposed mice, and its expression correlated with thermogenic capacity across adipose tissues (Fig. 2F), raising a question as to whether P33MONOX is required for thermogenic pentose cycling in BAT.

To probe the role of this pathway in BAT thermogenesis, we isolated BAT from mice housed at room temperature (22°C). Oxygen consumption was measured in the presence of a highly selective G6PD inhibitor (G6PDi) (*32*), serving as a proxy for mitochondrial substrate utilization and heat production (Fig. 2G). Inhibition of G6PD led to significantly reduced oxygen consumption, consistent with the hypothesis that glucose entry into the PPP is required for BAT thermogenesis. Next, to determine whether this pathway represents a key driver of increased glucose consumption or a compensatory response, we measured the effect of G6PD inhibition on basal and insulin-stimulated glucose uptake (Fig. 2H). BAT glucose uptake was not affected by G6PDi administration, suggesting that pentose cycling in isolation does not contribute to enhanced glucose consumption in cold-adapted BAT. Whether up-regulation of upper glycolysis is sufficient to mediate this effect is an important future research direction.

### Cold adaptation varies between adipose tissues

Despite being synonymous with cold exposure, proteomic changes in BAT (45% of proteome) were outnumbered by those in sWAT (62%; Fig. 1F). This raised the possibility that sWAT-specific changes might reflect the white adipose response to cold distinct from that associated with thermogenesis. Here, we sought to assess this hypothesis to determine how the molecular consequences of cold exposure differ between adipose tissue depots.

**Fig. 3.**
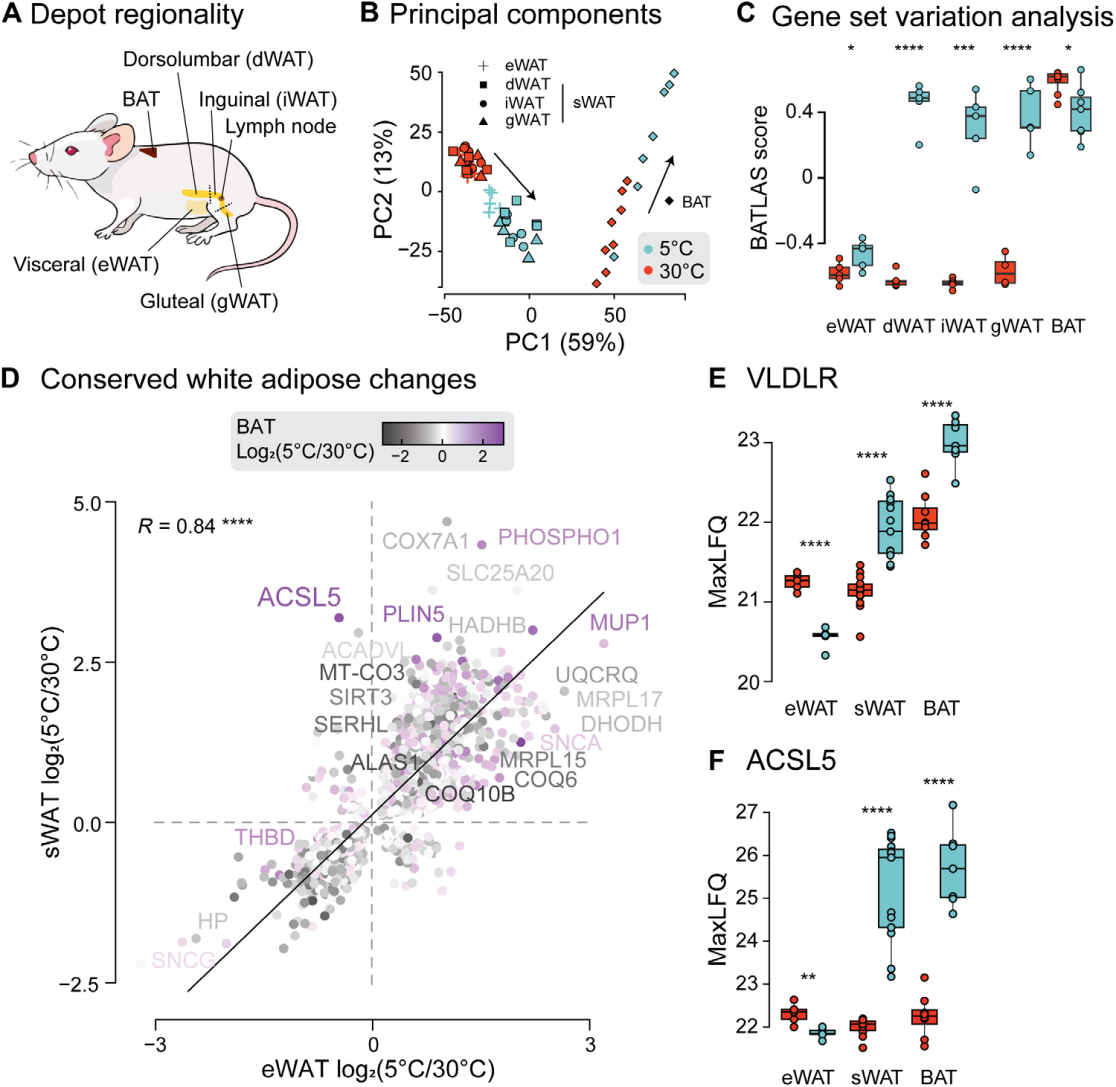
Cold adaptation varies between adipose tissues. (**A**) Depiction of subcutaneous adipose regions defined in relation to the central lymph node. (**B**) Samples from each adipose region are plotted in relation to the first two principal components. Arrows depict the cold-induced movement of each depot in the principal component space. Figures in brackets report the percentage of total variance explained by each principal component. (**C**) Results of gene set variation analysis showing the overall enrichment of 96 (of a total 119 reported) proteins associated with active BATs in the Brown Adipose Tissue Atlas (*77*). (**D**) Correlation between significantly altered proteins in eWAT and sWAT, colored by magnitude of change with cold exposure in BAT. Abundance of VLDLR (**E**) and ACSL5 (**F**) across depots. Significance was determined by a two-tailed Student’s *t* test accounting for multiple testing using an FDR correction. Significance is represented with *P < 0.05, **P < 0.01, ***P < 0.001, and ****P < 0.0001. *n* = 5 to 8 per condition.

Well-established differences in browning capacity have been described between visceral and subcutaneous adipose tissues, with subcutaneous adipose having greater thermogenic potential (*33*). Because heterogeneity has also been reported within the subcutaneous depot itself, depending on proximity to the lymph node (*34*), we further dissected sWAT into gluteal, inguinal, and dorsolumbar regions (Fig. 3A). Principal components analysis of adipose tissue proteomes revealed considerable overlap between eWAT and sWAT in mice housed under thermoneutral conditions, consistent with our previous study (*35*). However, sWAT exhibited more pronounced proteomic remodeling upon cold adaptation (Fig. 3B). Notably, BAT-specific remodeling was so pronounced that it captured the second principal component and explained 13% of the total variation between the proteomes (Fig. 3B), suggesting that proteomic adaptation to cold is fundamentally distinct between brown and white adipose tissues. No differences were detected between subcutaneous regions, indicating similar cold responses among all sWAT regions after 3 weeks of cold exposure. To assess the thermogenic capacity of each adipose region, gene set variation analysis was performed using 96 detected (of a total 119 reported) proteins associated with activated BAT. Consistent with total proteomic remodeling, this revealed stronger induction of the thermogenic program in sWAT compared to eWAT and an equivalent thermogenic response between sWAT regions (Fig. 3C). The decrease in BAT browning score following cold exposure was associated with significant down-regulation of 31 proteins. Most notably, this included PYGB (glycogen phosphorylase), BCKDHB (2-oxoisovalerate dehydrogenase), and ACADS (short-chain acyl-CoA dehydrogenase). Given that both glucose and branched chain amino acids are in high demand in cold-exposed BAT, decreased PYGB and BCKDHB may simply reflect decreased substrate availability after 3 weeks of adaptation. Down-regulation of ACADS may reflect decreased fatty acid oxidation as discussed above (Fig. 2B).

Despite greater induction of thermogenic proteins in sWAT, cold-induced remodeling was remarkably similar between visceral and subcutaneous adipose tissues at the level of the total proteome (Figs. 1F and 3D); 94% of proteins significantly regulated in both eWAT and sWAT were regulated consistently. This indicates that many changes in adipose tissues reflect the overall white adipose response to cold rather than changes required for thermogenesis. In line with the function of white adipose tissues to supply peripheral tissues with fatty acids, many of the cold-induced changes in both WAT tissues involved proteins that regulate fatty acid synthesis and release into circulation, including up-regulation of FASN and ACLY.

Closer inspection of the specific proteins regulated by cold, however, revealed unexpected intricacies in the response of different adipose tissue depots to cold (Fig. 3, E and F). For example, the very low density lipoprotein receptor (VLDLR), required for uptake of triglycerides (TGs) from the circulation, was down-regulated in eWAT but up-regulated in sWAT. This was also the case for fatty acid translocase (CD36), indicating that cold adaptation may enhance the uptake of triglycerides into thermogenic tissues by inhibiting their storage in eWAT. Further investigation of proteins uniquely remodeled in sWAT revealed proteins with potential roles in thermogenesis. One such candidate is acyl-CoA synthetase long chain family member 5 (ACSL5), which was significantly up-regulated in sWAT and BAT but not in eWAT (Fig. 3F). Although a relationship between ACSL5 and BAT activity was previously reported alongside LETMD1 (*36*), now considered to be an upstream regulator of UCP1 (*37*), a thermogenic role for ACSL5 has yet to be demonstrated experimentally.

### Thermokines synchronize metabolic adaptation

Coordinated changes in substrate preference and flux are likely central to the whole-body response to cold; however, it is unclear how these changes are synchronized between tissues. One possibility is that the metabolic requirements of each tissue might be communicated through the release of thermokines, temperature-regulated secreted factors, into the circulation. Here, we sought to assess the contribution of thermokines to the whole-body response to cold.

Analysis of the plasma proteome of cold-adapted mice reflected systemic metabolic changes. For example, plasma levels of PCSK9 (proprotein convertase subtilisin/kexin 9), a negative regulator of lipid entry into the cell (*38*), decreased by 80% in response to cold adaptation (Fig. 1F), consistent with increased lipid uptake into the liver, sWAT, and BAT. Elevated lipid oxidation increases the production of reactive oxygen species (*39*), and the most up-regulated plasma protein upon cold exposure was superoxide dismutase 3 (SOD3; Fig. 1F), best known for reducing levels of extracellular reactive oxygen species (*40*). SOD3 is secreted from BAT and quadriceps (*41*, *42*), and the levels of SOD3 in these tissues were also increased in our data, suggesting that it is possible to map changes in the plasma proteome to their tissue of origin.

**Fig. 4.**
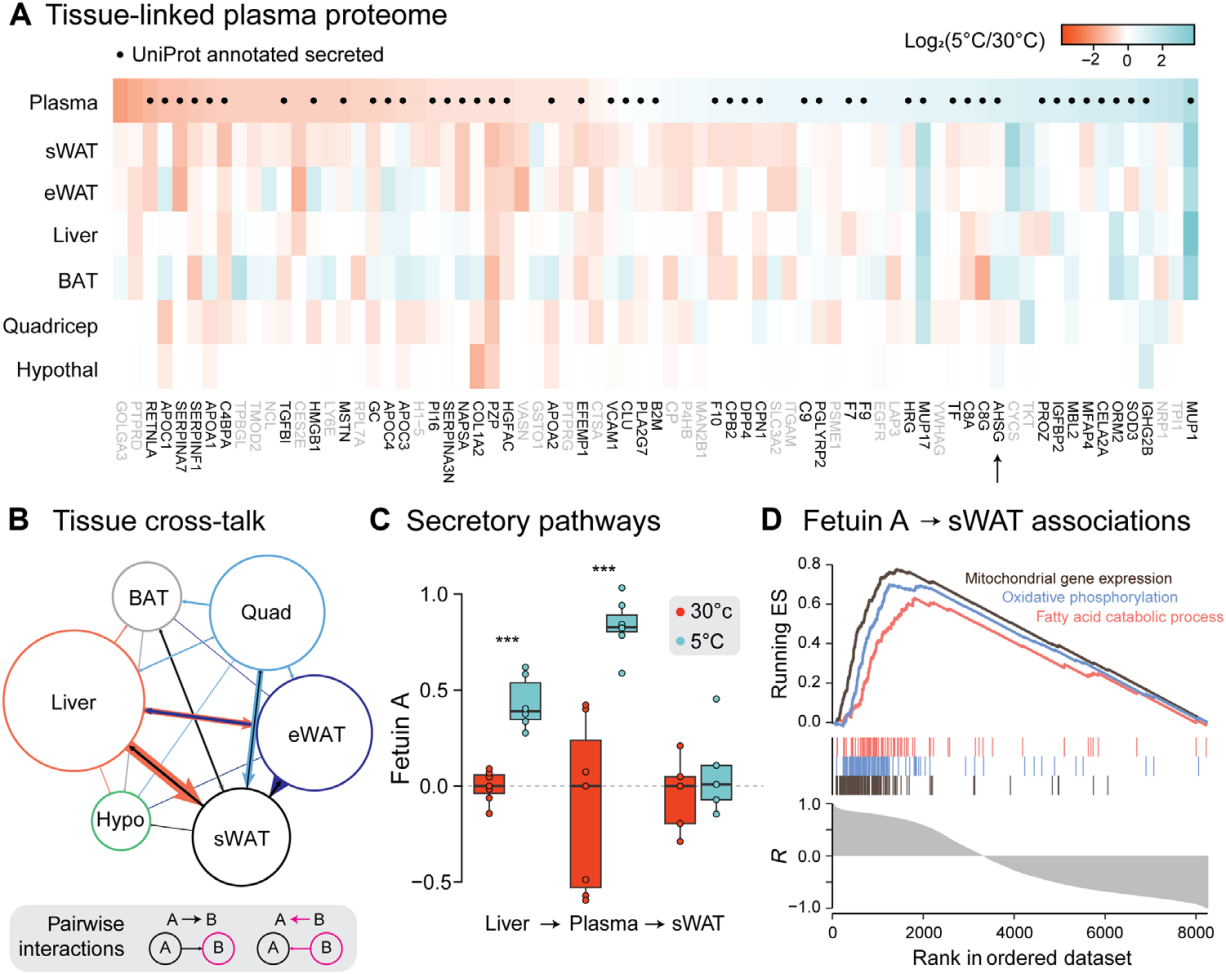
Thermokines synchronize metabolic adaptation. (**A**) Fold changes are reported across tissues for all plasma proteins significantly regulated by cold adaptation. Tissues are ordered by the number of plasma proteins significantly changed in each tissue. Proteins with a UniProt secreted annotation are labeled with a dot on the heatmap and with darker font for the name of the protein. AHSG (fetuin A), discussed in later panels, is highlighted using an arrow below the protein names. (**B**) Visualization of the strength of directional tissue cross-talk between pairs of tissues. The legend indicates the direction of tissue cross-talk. The width of each line is proportional to the strength of cross-talk, and the size of each circle is proportional to the strength of all cross-talk originating from that tissue. (**C**) Cold-induced fold changes in AHSG abundance across the liver, plasma, and sWAT, annotated with hypothesized model of action. (**D**) Top results of gene set enrichment for proteomic changes in sWAT that are predicted by liver AHSG abundance. Significance was determined using a two-tailed Student’s *t* test. Significance is represented with ***P < 0.001. *n* = 5 to 8 per condition.

Notably, we detected 73 plasma proteins that were significantly regulated by cold adaptation that were also detected in a tissue (Fig. 4A). A total of 60% of these was annotated as secretory proteins in UniProt. Next, we used an approach based on the Quantitative Endocrine Network Interaction Estimation pipeline (*43*) to enable pairwise comparisons of potential cross-talk between tissues. Briefly, this analysis identifies secreted proteins in one tissue that correlate with all other proteins in another tissue. This assumes that secreted proteins correlating with many cell processes in another tissue may do so because they directly signal the need for those changes. This identified the liver as a major site of release for thermokines, followed by quadriceps and eWAT (Fig. 4B). The strength of cross-talk from each tissue was independent of the number of proteins changed in that tissue following cold adaptation. For example, BAT exhibited little cross-talk with other organs despite exhibiting considerable cold-induced proteomic remodeling. BAT-derived secreted factors have previously been shown to have important roles in acute cold exposure (*17*), suggesting that a successful whole-body response might require precise timing of thermokine secretion from individual tissues. Here, we analyzed plasma after 3 weeks of cold exposure and so results are likely biased toward thermokines required for the maintenance of cold-induced metabolic rewiring rather than its initiation.

A major prediction of this approach was that many liver-derived thermokines appeared to target white adipose tissues. These predominantly appeared to target sWAT because changes in protein abundance in the liver were more closely correlated with the sWAT proteome than that of eWAT, suggesting a role for the liver in communicating systemic metabolic demands to sWAT. Candidate thermokines were ranked by known expression in the liver and the strength of predicted effects in sWAT, identifying alpha-2-HS glycoprotein (AHSG, also known as fetuin A) as a potential regulator of sWAT metabolic activity. Fetuin A is predominantly expressed in the liver, and so, the significant up-regulation in plasma following cold exposure is likely a direct consequence of increased production in the liver (Fig. 4C). Fetuin A abundance was not altered in sWAT. To identify putative functions for fetuin A, we performed gene set enrichment for sWAT proteins whose cold-induced changes could be predicted by fetuin A abundance in the liver (Fig. 4D). This indicates that fetuin A secreted from the liver might function to enhance sWAT thermogenesis by increasing lipid catabolism. Consistently, human genetic association studies have linked polymorphisms in fetuin A to altered lipolytic activity in subcutaneous adipose (*44*) and treatment of 3T3-L1 adipocytes with fetuin A stimulated a prolipolytic phenotype (*45*).

### Multitissue model of adaptive thermogenesis

**Fig. 5.**
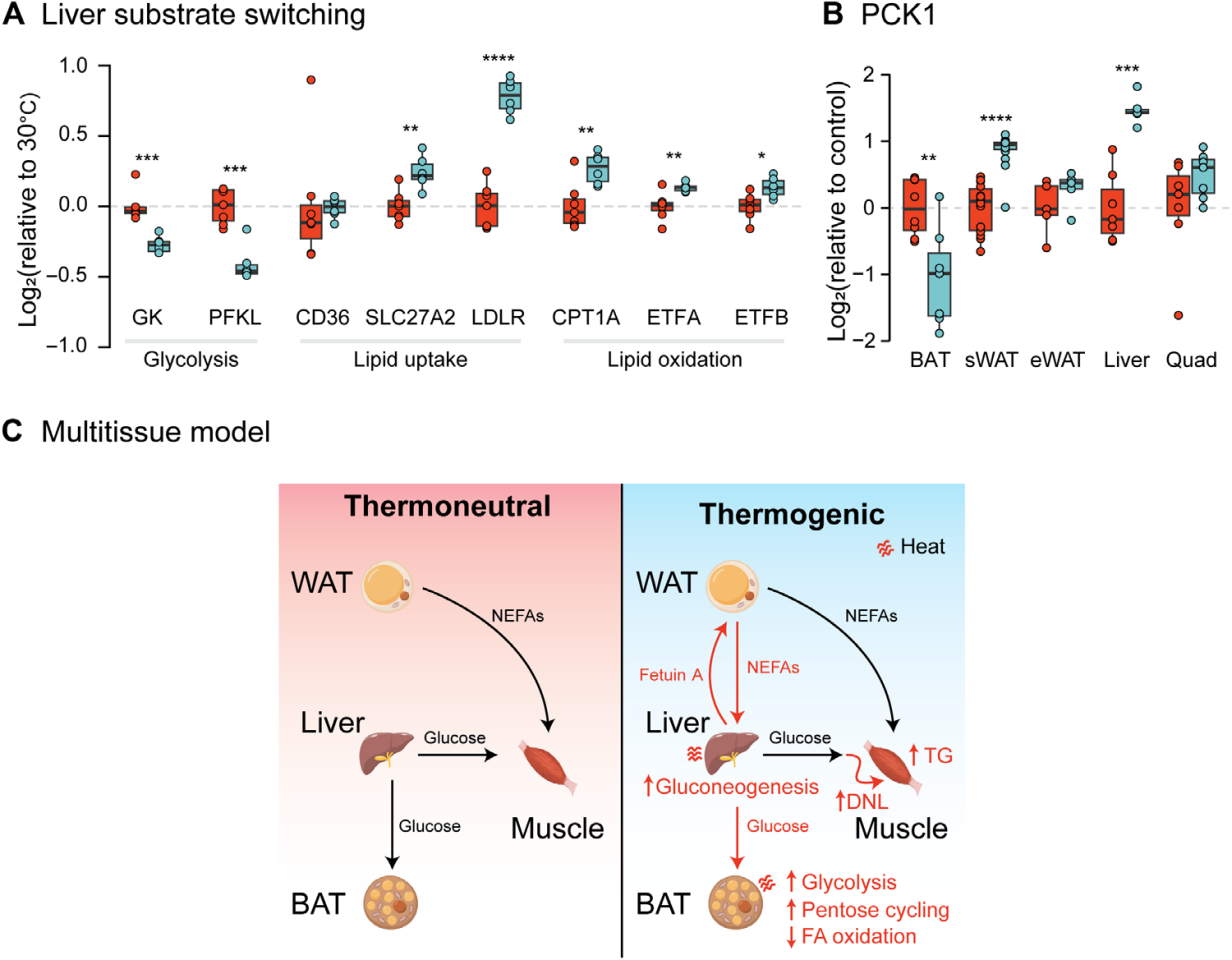
Multitissue model of adaptive thermogenesis. (**A**) Cold-induced changes in protein abundance in the liver for several central metabolic pathways, normalized to the thermoneutral mean. (**B**) Changes in protein abundance in the liver for proteins involved in gluconeogenesis and glucose metabolism. (**C**) Model schematic. Increased de novo lipogenesis (DNL) and release of nonesterified fatty acids (NEFAs) by WAT may provide energy for hepatic gluconeogenesis and glucose output and facilitate elevated glucose-dependent thermogenesis in BAT. Increased DNL in muscle likely acts to sequester fatty acids (FAs) for future use, resulting in elevated intramuscular lipid accumulation (*79*). TG, triglyceride. Significance was determined using a two-tailed Student’s *t* test. Significance is represented with *P < 0.05, **P < 0.01, ***P < 0.001, and ****P < 0.0001. *n* = 5 to 8 per condition.

To gain insight into the physiological consequences of cold adaptation, it is necessary to interpret tissue-specific changes in the context of the whole body. Considering changes across organs collectively, we hypothesize that successful cold adaptation relies on the establishment of a multitissue thermogenic pathway that is required for optimal BAT function. Consistent with this, the liver proteome underwent considerable remodeling (30% of proteome) after cold and, notably, changes in the abundance of glucose and fatty acid metabolic enzymes in BAT were reciprocated in the liver (Fig. 5A). For example, the levels of glucokinase and phosphofructokinase, both rate-limiting steps for glycolysis, were significantly down-regulated in cold adapted livers, suggesting reduced glycolytic flux (Fig. 5A) but up-regulated in BAT. In contrast, proteins involved in lipid uptake, including SLC27A2 (also known as FATP2) and LDLR, were markedly increased in the liver but decreased in BAT. Consistent with increased oxidation of fatty acids in the cold-adapted liver, CPT1A, ETFA, and ETFB were all significantly up-regulated. These observations suggest that cold adaptation leads to the adoption of a fatty acid preference in liver, opposite to that of BAT.

Together with the fact that circulating lipid species were also decreased by cold exposure, it is intriguing that the most significantly down-regulated proteins in the liver were enriched for enzymes involved in de novo lipogenesis (gene set enrichment analysis normalized enrichment score = −1.8; *P* < 0.05). Specifically, proteins such as ACLY, FASN, FABP5 (fatty acid binding protein 5), SCD1 (stearoyl-CoA desaturase), and ELOVL5 (hepatic fatty acid elongase 5) were down-regulated by 65 to 80% following cold adaptation, raising the possibility that de novo lipogenesis in the liver is shut off during cold adaption to conserve energy for glucose output and provision to other tissues such as BAT. Consistently, phosphoenolpyruvate carboxykinase 1 (PCK1), the rate-limiting enzyme of gluconeogenesis, was up-regulated 2.8-fold (Fig. 5B). Many enzymes involved in the internal metabolism of glucose, including GYS2 (glycogen synthase 2), PGM1 (phosphoglucomutase 1), and GBE1 (glycogen branching enzyme), were significantly decreased. The abundance of glycerol-3-phosphate dehydrogenase 2 (GPD2), which is required for gluconeogenesis and targeted by metformin (*46*), was 1.4-fold up-regulated in cold-adapted livers, consistent with increased glucose production. No changes were observed in liver glucose transporters.

Collectively, these changes in protein abundance suggest that the liver contributes to the whole-body response to cold by enhancing glucose production and reducing both its glucose storage and oxidation capacity. The utilization of fatty acids likely increases to compensate for reduced glucose oxidation, sparing glucose for tissues capable of thermogenesis. Together with results in BAT and insights from the plasma proteomic analysis, here we propose a multitissue model of adaptive thermogenesis (Fig. 5C).

### Cold adaptation and obesity have opposite effects on the brown adipose proteome

It is widely believed that the molecular pathways activated by cold exposure will have therapeutic utility in the treatment of obesity. While evidence exists to support this at a physiological level—for example, cold exposure triggers weight loss—it is yet to be proven that these interventions target equivalent mechanisms at the molecular level. To assess this, we generated BAT proteomes from lean and diet-induced obese C57BL/6J mice.

**Fig. 6.**
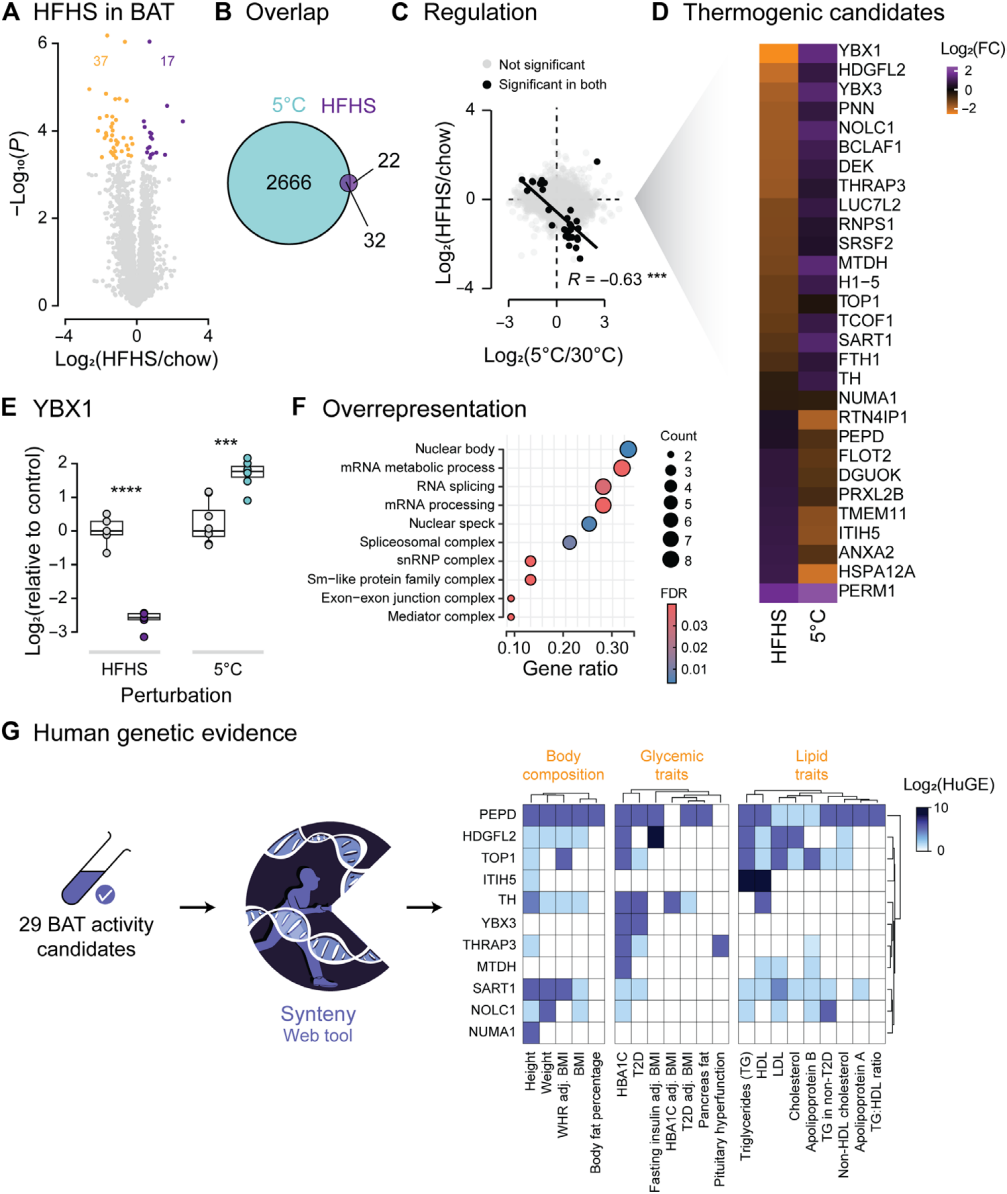
Cold adaptation and obesity have opposite effects on the brown adipose proteome. (**A**) Volcano plot comparing brown adipose proteomes of mice fed either a chow or HFHS diet for 6 weeks. Significantly regulated proteins are highlighted (FDR < 0.05). (**B**) Overlap of significantly regulated proteins between cold adaptation and HFHS feeding. (**C**) Correlation between fold changes induced by cold adaptation or HFHS feeding for significantly regulated proteins. (**D**) Heatmap demonstrating regulation of 32 overlapping proteins from (B). FC, fold change. (**E**) Change in YBX1 abundance induced by either HFHS feeding or cold adaptation in BAT. (**F**) Results of overrepresentation analysis for proteins differentially regulated by cold adaptation and HFHS feeding. (**G**) Heatmap of HuGE scores representing the strength of association between genes in (D) and body composition, glycemic traits, or lipid-related traits. Only genes with very strong associations (HuGE >30) to at least one trait are included. Heatmap generated using Synteny (*57*). BMI, body mass index. Significance was determined by a two-tailed Student’s *t* test accounting for multiple testing using an FDR correction. Significance is represented with ***P < 0.001 and ****P < 0.0001. *n* = 5 to 8 per condition.

High-fat, high-sugar (HFHS) feeding for 6 weeks elicited relatively few proteomic changes compared to cold exposure (Fig. 6A); however, intriguingly, almost 60% of BAT proteins altered by HFHS feeding were also affected by cold exposure (Fig. 6B). Most notably, more than 90% of these overlapping proteins were regulated in opposite directions in the two perturbations (Fig. 6, C and D). These data suggest that cold adaptation and HFHS feeding elicit opposite effects on the BAT proteome and, by likely extension, its function. This set of proteins was enriched for proteins that regulate RNA splicing (Fig. 6F). For example, YBX1 (Y box binding protein 1) (*47*, *48*), PNN (*49*), DEK (*50*, *51*), and ANXA2 (*52*) have all been implicated in splicing in various contexts. We also observed enrichment for the exon-exon junction, epitomized by neuronal protein tyrosine hydroxylase, which may suggest that neural innervation to BAT is altered by both obesogenic diets and cold exposure.

Consistent with the hypothesis that alterations to the BAT proteome induced by obesogenic diets might impair BAT thermogenesis, a thermogenic function has already been demonstrated for the most strongly differentially regulated protein, YBX1 (Fig. 6E). YBX1 regulates the commitment of subcutaneous adipocytes to a thermogenic lineage (*16*) and stimulates brown adipogenesis via enhanced RNA stability of transcripts required for mitophagy (*53*). Whether therapeutic benefit in the treatment of obesity might be obtained by targeting proteins such as YBX1 is an interesting question.

Many have argued that studying such processes will not identify useful therapeutic targets because many obese humans lack active BAT, although this continues to be actively debated (*54*, *55*). To explicitly assess the potential translational relevance of our findings, we leveraged the Human Genetic Evidence (HuGE) score framework to determine the extent to which genetic variation in the proteins identified above is associated with human metabolic disease (*56*). We used Synteny to obtain data for multiple genes simultaneously, a web tool we recently developed to enhance the accessibility of human genetic data (*57*). Consistent with the hypothesis that some of the proteins oppositely regulated by obesity and cold exposure are relevant to human health, 38% (11 of 29) of these proteins demonstrated very strong associations (HuGE ≥30) to one or more phenotypes related to human obesity (Fig. 6G). The strongest associated human phenotypes were hemoglobin subunit alpha 1C (HBA1C), type 2 diabetes, plasma triglycerides, and high-density lipoprotein cholesterol rather than obesity itself. This raises an interesting question as to whether the benefits of active BAT might relate to improved systemic metabolic health rather than a direct regulation of fat mass.

## DISCUSSION

Substantial efforts have been made to develop therapeutic approaches that mimic the thermogenic and weight-reducing effects of cold exposure, predominantly focusing on adipose tissues. Numerous studies have sought to identify key initiators of adipocyte browning and to understand how metabolism is rewired in response to cold exposure. Here, we present a multitissue proteomic resource to enable exploration of the mechanisms of cold adaptation beyond the isolated effects on individual adipose tissues. To advance the understanding of the molecular basis of cold-affected phenotypes, we have made all data publicly available through an interactive web tool (https://bigproteomics.shinyapps.io/ColdAdaptation/). These data strongly support the hypothesis that cold adaptation is a systemic process, highlighting the crucial role of downstream adaptations in the liver and white adipose tissue in sustaining BAT thermogenesis.

Considering how changes might evolve across tissues, it seems most likely that the whole-body response to cold originates in BAT where up-regulation of glycolytic enzymes and down-regulation of enzymes involved in fatty acid oxidation provide a mechanistic explanation for how BAT switches from fatty acid to glucose as its principal source of energy upon cold exposure. Next, a reciprocal switch in liver substrate preference from glucose (in thermoneutral mice) to fatty acids (in cold-exposed mice) appears to spare glucose for supply to BAT, with this metabolic state being characterized by an up-regulation of proteins involved in fatty acid oxidation and elevated PCK1, a hallmark of gluconeogenesis. Leveraging a unique approach to infer tissue cross-talk from plasma proteomic data, we identified major changes in the circulating levels of both fetuin A and PCSK9, which we propose are involved in coordinating enhanced release and uptake of fatty acids by the liver. Collectively, these studies highlight the utility of multitissue proteomic analysis to decipher complex physiological processes such as cold exposure.

### Pentose cycling in cold-activated BAT

The traditional view that fatty acids are the primary fuel source for BAT has recently been challenged by two independent ^13^C-glucose tracing studies that both found that cold-induced thermogenesis in BAT is primarily fueled by glucose (*9*, *10*). However, without some underlying mechanistic understanding, these reports have remained somewhat controversial. Here, we propose that enhanced glucose utilization in cold-adapted BAT is mediated by an up-regulation of proteins in upper and lower glycolysis and rewiring of the PPP. Despite increased expression of glucose-6-phosphate 1-dehydrogenase X (G6PDX), the rate-limiting step of entry into the PPP, enzymes involved in the nucleotide synthetic function of PPP, such as PRPS1, were significantly down-regulated or unchanged, suggesting that there is increased flux via the PPP to the level of R5P but not further. Consistent with pentose cycling metabolism, where R5P is shuttled back to glycolysis at the level of glyceraldehyde-3-phosphate by TKT, we observed up-regulation of TKT. To further probe the contribution of this metabolic rewiring to thermogenesis, we inhibited glucose entry into the PPP using a highly selective G6PDi, demonstrating a significant reduction in BAT oxygen consumption (Fig. 2G).

In neutrophils, where the pentose cycle was first described (*29*), the NADPH generated by this cycle is utilized by NOXs to fuel a characteristic superoxide burst required for neutrophil activation. Thus, we wondered whether a similar mechanism might also contribute to thermogenesis. Consistent with this hypothesis, the levels of both enzymes required for glutathione synthesis were significantly down-regulated by cold adaptation, suggesting that increasing the cytosolic concentration of superoxide may be critical to enabling maximal BAT thermogenesis. A functional role for UCP1 sulfenylation in UCP1 activation was previously reported by Chouchani *et al.* (*58*), with structural modeling predicting the reported Cys^253^ site to be inside the mitochondrial matrix. This would suggest that the mitochondria would be the likely source of superoxide for this step as a superoxide is relatively unstable (*59*). However, results from studies in isolated mitochondria demonstrate that UCP1 activity can be increased by increasing external superoxide concentrations (*30*). Thus, future studies are likely justified to determine whether these effects might be explained by sulfenylation of other cysteine residues in UCP1 or whether there are aspects of this model that remain to be clarified.

Although NOXs, such as NOX4, have previously been shown to regulate diet-induced obesity through impairments in BAT metabolic function (*60*), a clear thermogenic mechanism has not been described. In addition, no well-characterized NOXs were detected in our BAT proteome, suggesting that if they are expressed in these cells, they are likely to be low-abundance proteins. We observed cold-induced changes in the levels of a functionally uncharacterized NOX, P33MONOX, closely paralleled thermogenic differences across adipose depots, suggesting a role in thermogenesis. Thus, we hypothesize that increased glucose utilization by cold-adapted BAT stems from pentose cycling, leading to enhanced superoxide production via P33MONOX that ultimately activates UCP1 to bolster thermogenesis. Future studies should seek to investigate the functions of P33MONOX in knockdown or overexpression mouse models.

Although we did not observe changes in any lipogenic proteins, it is also important to note that increased NADPH levels may also lead to enhanced lipogenesis and that this may also enhance UCP1 activity (*61*, *62*). UCP1 contains a cytosol-facing fatty acid binding domain whose activity is proportional to the chain length of the bound fatty acid, and up-regulation of fatty acid elongases is a prominent feature of cold adaptation in BAT (*63*).

### Adaptations across tissues

Consistent with the hypothesis that enhanced hepatic gluconeogenesis is critical for BAT thermogenesis (*10*), reciprocal changes in protein expression were observed in cold-adapted BAT and liver. Mechanistically, this was underpinned by increased expression of PCK1, the rate-limiting gluconeogenic enzyme, as well as up-regulation of proteins involved in fatty acid oxidation. This suggests that enhanced fatty acid utilization in the liver enables glucose to be spared for output into the circulation. Many substrates for gluconeogenesis are three-carbon metabolites, and levels of GPD2, which facilitates the entry of glycerol into gluconeogenesis, were significantly increased in liver.

Enhanced lipolysis in white adipose tissue may provide fatty acids to fuel this process, because we also observed changes in lipolytic proteins in eWAT. Notably, circulating levels of fetuin A were increased during cold exposure, likely due to increased secretion from the liver. Fetuin A has been shown to induce lipolysis in adipocytes (*45*), raising the possibility of a multitissue metabolic network that enables animals to defend against the cold. Consistent with this model, tissues that supply substrates to one another exhibit opposite regulation of enzymes responsible for metabolizing those substrates. For example, proteins involved in de novo lipogenesis were increased in eWAT but decreased in the liver. Similarly, proteins involved in glucose oxidation were down-regulated in the liver but up-regulated in BAT. This is consistent with a multitissue model where, in each step of the pathway, elevated demand for particular substrates is met by increased supply from downstream tissues.

This model unifies several key processes observed in cold-adapted animals, including (i) increased lipolysis in white adipose tissue facilitated by increased lipogenesis, (ii) up-regulated hepatic gluconeogenesis, and (iii) enhanced glucose-dependent thermogenesis in BAT.

### Diet-induced obesity

By integrating data from cold-adapted mice with proteomic data from mice fed a HFHS diet, we observed that approximately half of the proteins regulated by diet-induced obesity in BAT were also regulated by cold adaptation. A total of 90% of these proteins was regulated in opposite directions. Consistent with the strong inverse relationship between BAT activity and obesity in humans, this indicated that an impairment of thermogenic processes in BAT may be critical to the obesogenic nature of HFHS diets. Proteins oppositely regulated by diet and cold exposure were strongly enriched for RNA splicing pathways, suggesting that posttranscriptional mechanisms may be critical to regulating BAT activity.

The role of RNA splicing and processing is quite well established for both adipogenesis and thermogenesis, meaning that there is precedent for this form of functional regulation in brown adipose (*64*–*66*). With the development of improved long-read sequencing platforms, it is increasingly possible to understand how the splicing of individual transcripts is regulated in disease states. Our findings suggest that by applying such technologies to BAT samples from individuals exposed to either cold or HFHS diets, it may be possible to identify specific transcripts that explain the development of obesity. Experimental characterization of the candidates presented here may also provide insight into how the BAT transcriptional program might be targeted to enhance energy expenditure to combat obesity.

Many of the top differentially regulated proteins have convincing genetic evidence for involvement in common metabolic diseases in humans (Fig. 6G). For example, hepatoma-derived growth factor–like 2, an epigenetic regulator (*67*), has a compelling association with fasting insulin concentrations. However, in contrast to our original hypothesis that the proteins identified by this analysis would associate with body composition phenotypes, we instead observed that most proteins associated with obesity-related complications, such as HBA1C and plasma triglycerides. This suggests that the translational benefit of active BAT might be to facilitate states of metabolically healthy obesity rather than in directly combating obesity. This has also been observed in human studies. For example, Herz *et al.* (*68*) reported that 35% of severely obese study participants had active BAT and, where this was observed, patients had significantly decreased HBA1C and insulin levels, consistent with the results of our analysis comparing cold-exposed and diet-induced obese mice. Thus, the results of this analysis provide potential insight into the molecular underpinnings of metabolically healthy obesity, arguably more therapeutically relevant than obesity itself (*69*).

### Limitations

The principal limitation of this study is that it relies upon tissue proteomics to infer function. In the human population, however, phenotypic diversity is largely driven by genetic variants that, for the most part, confer their phenotypic effects via modest changes in protein expression. Thus, to ensure that studying protein levels can provide deep insights into system behavior, we largely restricted our analysis to proteins that differed by more than twofold between groups. Although further studies will be required in the future to validate some of our findings, this study provides important clues about key regulatory pathways that contribute to the physiology of cold adaptation. In addition, tissues were analyzed after chronic cold acclimatization and so it is worth recognizing that this potentially misses dynamic changes in fuel utilization throughout the time course.

The study’s reliance on animal models also necessitates caution when extrapolating results to humans. Because many components of the whole-body response to cold may be deleterious in the thermoneutral state, the risks of activating thermogenesis-associated processes must be carefully considered in humans. For example, although we observed significant up-regulation of lipolytic proteins in white adipose tissues, elevated circulating fatty acid levels are a central risk factors for many metabolic diseases (*70*). Although our analysis identified AHSG (also known as fetuin A) as a thermogenic signal to boost lipid breakdown in adipose tissue, elevated fetuin A levels in humans are a significant risk factor for metabolism-associated fatty liver disease in humans (*71*, *72*). This may mean that enhancing lipolysis is only beneficial if there is an elevated need for fatty acids as is the case with cold exposure. Thus, preclinical testing of such therapies in thermoneutral settings will be essential to assessing their safety and potential complications.

The ability of Systems Biology to provide functional insights into the behavior of complex systems hinges on advances in omics techniques. In this study, we focused solely on proteomic analysis because of current limitations in metabolomics and lipidomics. This is unfortunate, as our interpretations would have been strengthened by inclusion of metabolite-level data, particularly for NADPH and glycolytic metabolites. However, the inherent instability of the metabolome presents a challenge in ensuring that frozen tissue samples from an animal accurately represent in vivo conditions. While studies in individual tissues may mitigate this issue, it remains a considerable limitation for multitissue studies where it is not feasible to prioritize specific tissues in advance. Thus, future studies should be dedicated to the development of enhanced tissue-collection methods that stabilize the metabolome, making it possible to obtain more reliable data from frozen samples. Although relatively little is currently known about the function of lipids and their interactions with particular proteins, this gap in understanding will likely narrow as more studies are performed and these relationships are clarified.

## MATERIALS AND METHODS

### Experimental design

For both cold exposure and HFHS feeding studies, 10-week-old male C57BL/6J mice were obtained from the Animal Resources Centre (Perth, WA, Australia). Mice were acclimatized to housing conditions and handling for 2 weeks before experimentation, maintained at 23°C on a 12-hour light/dark cycle and given ad libitum access to food and water in individually ventilated cages with a density of five mice per cage. All experiments were performed in accordance with NHMRC (Australia) guidelines under the approval of The University of Sydney Animal Ethics Committee (AEC 2024/2480). Mice were monitored twice per week and weighed weekly. Lean mass and adiposity were determined using an EchoMRI-900 (EchoMRI Corporation Ptd., Ltd.).

Mice involved in the cold exposure study were fed a standard laboratory chow diet containing 13% calories from fat, 65% calories from carbohydrate, and 22% calories from protein (“Irradiated Rat and Mouse Diet,” Specialty Feeds, Glen Forest, WA, Australia), while mice involved in the feeding study were randomized to either a chow diet or an HFHS diet made in-house containing 45% calories from fat, 35% calories from carbohydrate, and 20% calories from protein, manufactured to closely resemble D12451 (Research Diets, NJ). Specifically, the HFD diet contained 23% (w/w) casein, 0.3% (w/w) methionine, 2% (w/w) gelatine, 20.2% (w/w) sucrose, 17% (w/w) corn starch, 5% (w/w) bran, 3% (w/w) safflower oil, 22% (w/w) lard, 5.8% (w/w) AIN-93 mineral mix (MP Biomedicals), 0.4% (w/w) choline bitartrate, and 1.3% (w/w) AIN-93 vitamin mix (MP Biomedicals).

Mice were housed in thermochambers within the Core Research Animal Facility at the Charles Perkins Centre, University of Sydney. Thermoneutral mice were single housed at 30°C for 3 weeks with standard enrichment. Mice undergoing cold exposure were briefly acclimatized to the cold by single housing at 23°C for 1 day with minimal enrichment or insulative material (such as nesting). Following this, these remained single housed (to prevent warmth from huddling) for 3weeks. Humidity was maintained between 40 and 50% for both temperatures.

### Plasma lipid assays

Mice were fasted for 2 hours (0900 to 1100) before whole blood was collected via tail bleed. Briefly, the end of the tail was nicked using a scalpel and ~200 μl of blood was collected into an EDTA-coated microcentrifuge tube (Sarstedt) before being placed on ice. Blood samples were centrifuged at 2000*g* for 10 min at 4°C, and the plasma supernatant was then transferred to a clean tube. The concentrations of each lipid species were determined using the LabAssay Non-Esterified Fatty Acid Kit (FujiFilm), triglyceride reagent (Sigma-Aldrich), and Amplex Red Cholesterol Assay Kit (Thermo Fisher Scientific) for nonesterified fatty acids (NEFAs), triglycerides, and cholesterol, respectively. Assays were performed using 5 μl of plasma and according to the manufacturer’s instructions, calculating sample concentrations using a standard curve.

### Proteomic sample preparation

Mice were euthanized by cervical dislocation, and tissues were snap frozen in liquid nitrogen before being homogenized using a liquid nitrogen–cooled mortar and pestle. Tissues were stored at −80°C for subsequent analysis. Tissues were weighed and resuspended in 200 μl of 2% sodium deoxycholate (SDC) in 100 mM tris (pH 8.5) buffer before being boiled at 95°C for 10 min and lysed by bead beating using a Tissue Lyzer for a 1-min cycle at 30 Hz. Lysates were centrifuged at 13,000*g* for 10 min at 4°C, and the lysate was transferred to clean 2-ml tubes while avoiding fat cake. The remaining lipid was removed by chloroform methanol extraction. Six hundred microliters of methanol, 300 μl of chloroform, and 450 μl of water were added in sequence with brief vortexing between each addition. Samples were centrifuged at 9000*g* for 5 min at 4°C to achieve phase separation. The upper layer was mostly removed, leaving about 50 μl to not disturb the protein pellet. Six hundred microliters of methanol was added, samples were centrifuged at 18,000*g* for 5 min at 4°C to pellet the protein, and the entire supernatant was removed. The protein pellet was resuspended in 60 μl of 2% SDC in 100 mM tris (pH 8.5). The protein concentration was determined by bicinchoninic acid assay. Ten micrograms of protein was aliquoted into 1.5-ml centrifuge tubes and adjusted to 1% SDC with H_2_O before reduction/alkylation buffer [10 mM tris(2-carboxyethyl)phosphine (TCEP) and 40 mM 2-chloroacetamide] was added, and the samples were heated for 10 min at 60°C. Once cooled to room temperature, 0.2 mg of trypsin and 0.2 mg of LysC were added to each sample and incubated overnight (18 hours) at 37°C with gentle agitation. An equal volume of 1% trifluoroacetic acid (TFA) in ethyl acetate was added to each sample to stop digestion and remove SDC.

Sample preparation for plasma proteomics was performed using two methods to increase proteomic depth. The first method was performed using undepleted plasma as follows. Frozen plasma was defrosted on ice, and 1 μl of plasma was added to 24 μl of SDC buffer [1% SDC, 100 mM tris-HCl (pH 8.5), 40 mM chloroacetamide, and 10 mM tris(2-carboxyethyl)phosphine] and heated to 95°C for 30 min with shaking to denature, reduce, and alkylate samples. Once cooled, samples were diluted 10-fold with 100 mM tris-HCl (pH 8.5) and digested as above. The second method was performed according to the small protein enrichment assay (SPEA) (*73*).

Tissue and plasma samples were then prepared for mass spectrometry analysis by StageTip cleanup using SDB-RPS solid phase extraction material (*74*). Briefly, three layers of SDB-RPS material were packed into 200-μl tips and 200 μl of samples was loaded onto StageTips by centrifugation at 1000*g* for 5 min. Stage tips were washed with subsequent spins at 1000*g* for 5 min with 100 μl of 1% TFA in ethyl acetate, then 1% TFA in isopropanol, and 0.2% TFA in 5% acetonitrile (ACN). Samples were eluted by addition of 100 μl of 60% ACN with 5% NH_4_OH. Samples were dried by vacuum centrifugation and reconstituted in 20 μl of 5% formic acid.

### Proteomic analysis

Samples were analyzed using a Dionex UltiMate 3000 RSLCnano LC coupled to a Q-Exactive HFX mass spectrometer (Thermo Fisher Scientific). One microgram of peptide sample was injected onto an in-house packed column (75 μm by 55 cm; 1.9-μm particle size, ReproSil Pur C18-AQ) and separated using gradient elution, with buffer A consisting of 0.1% formic acid in water and buffer B consisting of 0.1% formic acid in 80% ACN. Tissue samples were loaded to the column at a flow rate of 0.5 ml/min at 100% buffer A for 12 min before ramping to 19% buffer B over 80 min and then to 98% buffer B over 40 min and held for 10 min. Plasma samples were loaded as previously described (*73*) using a 60-min gradient. Eluting peptides were ionized by electrospray with a spray voltage of 2.4 kV and a transfer capillary temperature of 300°C. Mass spectra were collected using a data-independent acquisition (DIA) method with varying isolation width windows [widths of 27 to 589 mass/charge ratio (*m*/*z*)] between 350 and 1650 according to Table 1. MS1 spectra were collected between *m*/*z* 350 and 1650 at a resolution of 120,000. Ions were fragmented with a higher-energy collisional dissociation collision energy at 25%, and MS2 spectra were collected between *m*/*z* 300 and 2000 at a resolution of 30,000, with an automatic gain control target of 3 × 10^6^ and the maximum injection time set to automatic.

**Table 1.** Variable width isolation windows for mass spectrometry method.

DIA window	Min	Max	*m*/*z* center	Window width
1	350	394	372.0	44
2	393	424	408.5	31
3	423	452	437.5	29
4	451	478	464.5	27
5	477	504	490.5	27
6	503	529	516.0	26
7	528	555	541.5	27
8	554	581	567.5	27
9	580	608	594.0	28
10	607	635	621.0	28
11	634	663	648.5	29
12	662	693	677.5	31
13	692	725	708.5	33
14	724	759	741.5	35
15	758	798	778.0	40
16	797	841	819.0	44
17	840	892	866.0	52
18	891	959	925.0	68
19	958	1062	1010.0	104
20	1061	1650	1355.5	589

### Proteomic data processing

Proteomic raw data files were searched using DIA-NN using a library free FASTA search against the reviewed UniProt mouse proteome (downloaded December 2023) with deep learning enabled (*75*). The protease was set to trypsin/P with one missed cleavage, N-term methionine excision, and carbamidomethylation and methionine oxidation options on. The peptide length was set to 7 to 30, the precursor range to 350 to 1650, and the fragment range to 300 to 2000, and FDR was set to 1%.

To increase proteomic depth, plasma results from the two sample processing methods were combined by supplementing results from the SPEA with proteins uniquely detected in undepleted plasma. Where proteins were detected using both methods, only SPEA data were retained for downstream analysis. The final plasma proteomic dataset contained 558 proteins identified using SPEA and 243 proteins from undepleted plasma.

### BAT oxygen consumption

High-resolution respirometry experiments were conducted in C57BL/6J BAT using the OROBOROS Oxygraph-O2K (Oroboros Instruments, Corp., Innsbruck, AT). Mice were euthanized by cervical dislocation before BATs were dissected and placed in BIOPS preservation solution on ice [50 mM K^+^-MES, 20 mM taurine, 0.5 mM dithiothreitol, 6.56 mM MgCl_2_, 5.77 mM adenosine 5′-triphosphate, 15 mM phosphocreatine, 20 mM imidazole (pH 7.1) adjusted with 5 M KOH at 0°C, and 10 mM Ca-EGTA buffer (2.77 mM CaK_2_EGTA + 7.23 mM K_2_EGTA; 0.1 μM free calcium)]. Before addition of the samples to the OROBOROS chamber, tissues were washed three times in MiR05 buffer [110 mM sucrose, 60 mM K^+^-lactobionate, 0.5 mM EGTA, 3 mM MgCl_2_, 20 mM taurine, 10 mM KH_2_PO_4_, 20 mM Hepes, and bovine serum albumin (BSA; 1 g liter^−1^) essentially fatty acid free, pH 7.1 with KOH at 37°C]. The OROBOROS chambers contained 2 ml of MiR05, stirred at a constant speed of 12.5 Hz (750 rpm) at 37°C. Oxygen levels were maintained between 350 and 400 μM. A scalpel was used to further dissect BAT into ~2-mg pieces in ice-cold MiR05 buffer. These were added to the respirometry chamber without tissue permeabilization, and O_2_ concentration and O_2_ consumption (JO_2_) were recorded. Either dimethyl sulfoxide (DMSO) or G6PDi (*32*) dissolved in DMSO was titrated into the closed chambers using a Hamilton syringe.

### BAT glucose uptake

Adipose tissue was minced using scissors and basalled at 37°C for 2 hours in Dulbecco’s modified Eagle’s medium with 2% BSA and 25 mM Hepes. The explants were washed into prewarmed KRP [0.6 mM Na_2_HPO_4_, 0.4 mM NaH_2_PO_4_, 120 mM NaCl, 6 mM KCl, 1 mM CaCl_2_, 1.2 mM MgSO_4_, and 25 mM Hepes (pH 7.4)] with 2% BSA. Either DMSO or G6PDi was added for 30 min, and then insulin was added for a further 20 min. [^3^H]-2DG uptake was assessed during the past 5 min of the 20-min incubation. [^14^C]-mannitol was used to correct for extracellular [^3^H]-2DG. Tissue was lysed with 100 mM NaOH, and radiation was detected in a liquid scintillation counter. Each sample was normalized to its DNA amount by adding SYBR green and measuring fluorescence on a plate reader.

### Statistical analyses

All analysis and data visualization were conducted using the R programming environment. Unpaired two-tailed Student’s *t* tests were performed to determine significance, and *P* values were adjusted using a false discovery rate (FDR) correction. A note is made where other statistical tests are performed. Significance is represented with *P < 0.05, **P < 0.01, ***P < 0.001, and ****P < 0.0001.

Gene set variation analysis for adipose tissues was performed using the GSVA package (*76*) with the brown adipose gene set reported here (*77*). Gene set enrichment and overrepresentation analyses were performed using the clusterProfiler package (*78*). In overrepresentation tests, the tissue proteome was used as the background to correct for any enrichment related to the intrinsic function of each tissue.

Analysis of thermokines was based on the Quantitative Endocrine Network Interaction Estimation pipeline (*43*). Briefly, pairwise correlation analysis was performed to correlate every protein between each possible pair of tissues. Proteins were scored on the basis of the number of significantly correlating proteins in each other tissue, and these scores were summed to provide a directional score between each pair of tissues.

## References

[1] C. Huo, Z. Song, J. Yin, Y. Zhu, X. Miao, H. Qian, J. Wang, L. Ye, L. Zhou, Effect of acute cold exposure on energy metabolism and activity of brown adipose tissue in humans: A systematic review and meta-analysis. Front. Physiol. 13, 917084 (2022). 35837014 10.3389/fphys.2022.917084 PMC9273773

[2] T. J. Saari, J. Raiko, M. U-Din, T. Niemi, M. Taittonen, J. Laine, N. Savisto, M. Haaparanta-Solin, P. Nuutila, K. A. Virtanen, Basal and cold-induced fatty acid uptake of human brown adipose tissue is impaired in obesity. Sci. Rep. 10, 14373 (2020). 32873825 10.1038/s41598-020-71197-2 PMC7463032

[3] A. L. Vallerand, I. Jacobs, Influence of cold exposure on plasma triglyceride clearance in humans. Metabolism 39, 1211–1218 (1990). 2233284 10.1016/0026-0495(90)90097-v

[4] P. Lee, S. Smith, J. Linderman, A. B. Courville, R. J. Brychta, W. Dieckmann, C. D. Werner, K. Y. Chen, F. S. Celi, Temperature-acclimated brown adipose tissue modulates insulin sensitivity in humans. Diabetes 63, 3686–3698 (2014). 24954193 10.2337/db14-0513 PMC4207391

[5] M. J. W. Hanssen, J. Hoeks, B. Brans, A. A. J. J. Van Der Lans, G. Schaart, J. J. Van Den Driessche, J. A. Jörgensen, M. V. Boekschoten, M. K. C. Hesselink, B. Havekes, S. Kersten, F. M. Mottaghy, W. D. Van Marken Lichtenbelt, P. Schrauwen, Short-term cold acclimation improves insulin sensitivity in patients with type 2 diabetes mellitus. Nat. Med. 21, 863–865 (2015). 26147760 10.1038/nm.3891

[6] A. Hruby, F. B. Hu, The epidemiology of obesity: A big picture. Pharmacoeconomics 33, 673–689 (2015). 25471927 10.1007/s40273-014-0243-x PMC4859313

[7] M. Saito, Y. Okamatsu-Ogura, M. Matsushita, K. Watanabe, T. Yoneshiro, J. Nio-Kobayashi, T. Iwanaga, M. Miyagawa, T. Kameya, K. Nakada, Y. Kawai, M. Tsujisaki, High incidence of metabolically active brown adipose tissue in healthy adult humans: Effects of cold exposure and adiposity. Diabetes 58, 1526–1531 (2009). 19401428 10.2337/db09-0530 PMC2699872

[8] B. Cannon, J. Nedergaard, Brown adipose tissue: Function and physiological significance. Physiol. Rev. 84, 277–359 (2004). 14715917 10.1152/physrev.00015.2003

[9] G. Park, J. A. Haley, J. Le, S. M. Jung, T. P. Fitzgibbons, E. D. Korobkina, H. Li, S. M. Fluharty, Q. Chen, J. B. Spinelli, C. M. Trivedi, C. Jang, D. A. Guertin, Quantitative analysis of metabolic fluxes in brown fat and skeletal muscle during thermogenesis. Nat. Metab. 5, 1204–1220 (2023). 37337122 10.1038/s42255-023-00825-8 PMC10696589

[10] M. R. Bornstein, M. D. Neinast, X. Zeng, Q. Chu, J. Axsom, C. Thorsheim, K. Li, M. C. Blair, J. D. Rabinowitz, Z. Arany, Comprehensive quantification of metabolic flux during acute cold stress in mice. Cell Metab. 35, 2077–2092.e6 (2023). 37802078 10.1016/j.cmet.2023.09.002 PMC10840821

[11] A. Grefhorst, J. C. van den Beukel, W. Dijk, J. Steenbergen, G. J. Voortman, S. Leeuwenburgh, T. J. Visser, S. Kersten, E. C. H. Friesema, A. P. N. Themmen, J. A. Visser, Multiple effects of cold exposure on livers of male mice. J. Endocrinol. 238, 91–106 (2018). 29743343 10.1530/JOE-18-0076

[12] M. J. Betz, S. Enerbäck, Targeting thermogenesis in brown fat and muscle to treat obesity and metabolic disease. Nat. Rev. Endocrinol. 14, 77–87 (2018). 29052591 10.1038/nrendo.2017.132

[13] S. H. Kim, J. Plutzky, Brown fat and browning for the treatment of obesity and related metabolic disorders. Diabetes Metab. J. 40, 12–21 (2016). 26912151 10.4093/dmj.2016.40.1.12 PMC4768046

[14] J. Mukherjee, A. Baranwal, K. N. Schade, Classification of therapeutic and experimental drugs for brown adipose tissue activation: Potential treatment strategies for diabetes and obesity. Curr. Diabetes Rev. 12, 414–428 (2016). 27183844 10.2174/1573399812666160517115450 PMC5425649

[15] R. B. Burl, E. A. Rondini, H. Wei, R. Pique-Regi, J. G. Granneman, Deconstructing cold-induced brown adipocyte neogenesis in mice. eLife 11, e80167 (2022). 35848799 10.7554/eLife.80167 PMC9348851

[16] A. Rabiee, K. Plucińska, M. S. Isidor, E. L. Brown, M. Tozzi, S. Sidoli, P. S. S. Petersen, M. Agueda-Oyarzabal, S. B. Torsetnes, G. N. Chehabi, M. Lundh, A. Altıntaş, R. Barrès, O. N. Jensen, Z. Gerhart-Hines, B. Emanuelli, White adipose remodeling during browning in mice involves YBX1 to drive thermogenic commitment. Mol. Metab. 44, 101137 (2021). 33285300 10.1016/j.molmet.2020.101137 PMC7779825

[17] C. Scheele, C. Wolfrum, Brown adipose crosstalk in tissue plasticity and human metabolism. Endocr. Rev. 41, 53–65 (2020). 31638161 10.1210/endrev/bnz007 PMC7006230

[18] D. Carper, M. Coué, E. B. M. Nascimento, V. Barquissau, D. Lagarde, C. Pestourie, C. Laurens, J. V. Petit, M. Soty, L. Monbrun, M.-A. Marques, Y. Jeanson, Y. Sainte-Marie, A. Mairal, S. Déjean, G. Tavernier, N. Viguerie, V. Bourlier, F. Lezoualc’h, A. Carrière, W. H. M. Saris, A. Astrup, L. Casteilla, G. Mithieux, W. van Marken Lichtenbelt, D. Langin, P. Schrauwen, C. Moro, Atrial natriuretic peptide orchestrates a coordinated physiological response to fuel non-shivering thermogenesis. Cell Rep. 32, 108075 (2020). 32846132 10.1016/j.celrep.2020.108075

[19] N. Hadadi, M. Spiljar, K. Steinbach, M. Çolakoğlu, C. Chevalier, G. Salinas, D. Merkler, M. Trajkovski, Comparative multi-tissue profiling reveals extensive tissue-specificity in transcriptome reprogramming during thermal adaptation. eLife 11, e78556 (2022). 35578890 10.7554/eLife.78556 PMC9113744

[20] P. Flachs, K. Adamcova, P. Zouhar, C. Marques, P. Janovska, I. Viegas, J. G. Jones, K. Bardova, M. Svobodova, J. Hansikova, O. Kuda, M. Rossmeisl, U. Liisberg, A. G. Borkowska, K. Kristiansen, L. Madsen, J. Kopecky, Induction of lipogenesis in white fat during cold exposure in mice: Link to lean phenotype. Int. J. Obes. 41, 372–380 (2016). 10.1038/ijo.2016.228 28008171

[21] L. J. Bukowiecki, A. Géloën, A. J. Collet, Proliferation and differentiation of brown adipocytes from interstitial cells during cold acclimation. Am. J. Physiol. 250, C880–C887 (1986). 3717329 10.1152/ajpcell.1986.250.6.C880

[22] J. Nedergaard, Y. Wang, B. Cannon, Cell proliferation and apoptosis inhibition: Essential processes for recruitment of the full thermogenic capacity of brown adipose tissue. Biochim. Biophys. Acta Mol. Cell Biol. Lipids 1864, 51–58 (2019). 29908367 10.1016/j.bbalip.2018.06.013

[23] K. Almind, M. Manieri, W. I. Sivitz, S. Cinti, C. R. Kahn, Ectopic brown adipose tissue in muscle provides a mechanism for differences in risk of metabolic syndrome in mice. Proc. Natl. Acad. Sci. U.S.A. 104, 2366–2371 (2007). 17283342 10.1073/pnas.0610416104 PMC1892979

[24] W. Wu, F. Shi, D. Liu, R. P. Ceddia, R. Gaffin, W. Wei, H. Fang, E. D. Lewandowski, S. Collins, Enhancing natriuretic peptide signaling in adipose tissue, but not in muscle, protects against diet-induced obesity and insulin resistance. Sci. Signal. 10, eaam6870 (2017). 28743802 10.1126/scisignal.aam6870 PMC7418652

[25] S. Wilson, P. L. Thurlby, J. R. S. Arch, Substrate supply for thermogenesis induced by the β-adrenoceptor agonist BRL 26830A. Can. J. Physiol. Pharmacol. 65, 113–119 (1987). 2882828 10.1139/y87-023

[26] B. T. McNeill, N. M. Morton, R. H. Stimson, Substrate utilization by brown adipose tissue: What’s hot and what’s not? Front Endocrinol (Lausanne) 11, 571659 (2020). 33101206 10.3389/fendo.2020.571659 PMC7545119

[27] S. M. Furler, G. J. Cooney, B. D. Hegarty, M. Y. Lim-Fraser, E. W. Kraegen, N. D. Oakes, Local factors modulate tissue-specific NEFA utilization: Assessment in rats using 3H-(R)-2-bromopalmitate. Diabetes 49, 1427–1433 (2000). 10969825 10.2337/diabetes.49.9.1427

[28] V. Panic, S. Pearson, J. Banks, T. S. Tippetts, J. N. Velasco-Silva, S. Lee, J. Simcox, G. Geoghegan, C. Bensard, T. van Ry, W. L. Holland, S. A. Summers, J. Cox, G. S. Ducker, J. Rutter, C. J. Villanueva, Mitochondrial pyruvate carrier is required for optimal brown fat thermogenesis. eLife 9, e52558 (2020). 32795388 10.7554/eLife.52558 PMC7476754

[29] E. C. Britt, J. Lika, M. A. Giese, T. J. Schoen, G. L. Seim, Z. Huang, P. Y. Lee, A. Huttenlocher, J. Fan, Switching to the cyclic pentose phosphate pathway powers the oxidative burst in activated neutrophils. Nat. Metab. 4, 389–403 (2022). 35347316 10.1038/s42255-022-00550-8 PMC8964420

[30] K. S. Echtay, D. Roussel, J. St-Plerre, M. B. Jekabsons, S. Cadenas, J. A. Stuart, J. A. Harper, S. J. Roebuck, A. Morrison, S. Pickering, J. C. Clapham, M. D. Brand, Superoxide activates mitochondrial uncoupling proteins. Nature 415, 96–99 (2002). 11780125 10.1038/415096a

[31] K. S. Echtay, Mitochondrial uncoupling proteins—What is their physiological role? Free Radic. Biol. Med. 43, 1351–1371 (2007). 17936181 10.1016/j.freeradbiomed.2007.08.011

[32] J. M. Ghergurovich, J. C. García-Cañaveras, J. Wang, E. Schmidt, Z. Zhang, T. TeSlaa, H. Patel, L. Chen, E. C. Britt, M. Piqueras-Nebot, M. C. Gomez-Cabrera, A. Lahoz, J. Fan, U. H. Beier, H. Kim, J. D. Rabinowitz, A small molecule G6PD inhibitor reveals immune dependence on pentose phosphate pathway. Nat. Chem. Biol. 16, 731–739 (2020). 32393898 10.1038/s41589-020-0533-x PMC7311271

[33] R. Jia, X.-Q. Luo, G. Wang, C.-X. Lin, H. Qiao, N. Wang, T. Yao, J. L. Barclay, J. P. Whitehead, X. Luo, J.-Q. Yan, Characterization of cold-induced remodelling reveals depot-specific differences across and within brown and white adipose tissues in mice. Acta Physiol. (Oxf.) 217, 311–324 (2016). 27064138 10.1111/apha.12688

[34] C. Barreau, E. Labit, C. Guissard, J. Rouquette, M. L. Boizeau, S. G. Koumassi, A. Carrière, Y. Jeanson, S. Berger-Müller, C. Dromard, F. Plouraboué, L. Casteilla, A. Lorsignol, Regionalization of browning revealed by whole subcutaneous adipose tissue imaging. Obesity 24, 1081–1089 (2016). 26999447 10.1002/oby.21455

[35] S. Madsen, M. E. Nelson, V. Deshpande, S. J. Humphrey, K. C. Cooke, A. Howell, A. Diaz-Vegas, J. G. Burchfield, J. Stöckli, D. E. James, Deep proteome profiling of white adipose tissue reveals marked conservation and distinct features between different anatomical depots. Mol. Cell. Proteomics 22, 100508 (2023). 36787876 10.1016/j.mcpro.2023.100508 PMC10014311

[36] A. Park, K.-E. Kim, I. Park, S. H. Lee, K.-Y. Park, M. Jung, X. Li, M. B. Sleiman, S. J. Lee, D.-S. Kim, J. Kim, D.-S. Lim, E.-J. Woo, E. W. Lee, B. S. Han, K.-J. Oh, S. C. Lee, J. Auwerx, J. Y. Mun, H.-W. Rhee, W. K. Kim, K.-H. Bae, J. M. Suh, Mitochondrial matrix protein LETMD1 maintains thermogenic capacity of brown adipose tissue in male mice. Nat. Commun. 14, 3746 (2023). 37353518 10.1038/s41467-023-39106-z PMC10290150

[37] H. Xiao, L. H. M. Bozi, Y. Sun, C. L. Riley, V. M. Philip, M. Chen, J. Li, T. Zhang, E. L. Mills, M. P. Emont, W. Sun, A. Reddy, R. Garrity, J. Long, T. Becher, L. P. Vitas, D. Laznik-Bogoslavski, M. Ordonez, X. Liu, X. Chen, Y. Wang, W. Liu, N. Tran, Y. Liu, Y. Zhang, A. M. Cypess, A. P. White, Y. He, R. Deng, H. Schöder, J. A. Paulo, M. P. Jedrychowski, A. S. Banks, Y.-H. Tseng, P. Cohen, L. T. Tsai, E. D. Rosen, S. Klein, M. Chondronikola, F. E. McAllister, N. Van Bruggen, E. L. Huttlin, B. M. Spiegelman, G. A. Churchill, S. P. Gygi, E. T. Chouchani, Architecture of the outbred brown fat proteome defines regulators of metabolic physiology. Cell 185, 4654–4673.e28 (2022). 36334589 10.1016/j.cell.2022.10.003 PMC10040263

[38] A. S. Peterson, L. G. Fong, S. G. Young, PCSK9 function and physiology. J. Lipid Res. 49, 1152–1156 (2008). 18375913 10.1194/jlr.E800008-JLR200 PMC2386899

[39] L.-J. Su, J.-H. Zhang, H. Gomez, R. Murugan, X. Hong, D. Xu, F. Jiang, Z.-Y. Peng, Reactive oxygen species-induced lipid peroxidation in apoptosis, autophagy, and ferroptosis. Oxid. Med. Cell. Longev. 2019, 5080843 (2019). 31737171 10.1155/2019/5080843 PMC6815535

[40] Y. Wang, R. Branicky, A. Noë, S. Hekimi, Superoxide dismutases: Dual roles in controlling ROS damage and regulating ROS signaling. J. Cell Biol. 217, 1915–1928 (2018). 29669742 10.1083/jcb.201708007 PMC5987716

[41] D. Gao, S. Hu, X. Zheng, W. Lin, J. Gao, K. Chang, D. Zhao, X. Wang, J. Zhou, S. Lu, H. R. Griffiths, J. Liu, SOD3 is secreted by adipocytes and mitigates high-fat diet-induced obesity, inflammation, and insulin resistance. Antioxid. Redox Signal. 32, 193–212 (2020). 31680537 10.1089/ars.2018.7628

[42] M. Okutsu, J. A. Call, V. A. Lira, M. Zhang, J. A. Donet, B. A. French, K. S. Martin, S. M. Peirce-Cottler, C. M. Rembold, B. H. Annex, Z. Yan, Extracellular superoxide dismutase ameliorates skeletal muscle abnormalities, cachexia, and exercise intolerance in mice with congestive heart failure. Circ. Heart Fail. 7, 519–530 (2014). 24523418 10.1161/CIRCHEARTFAILURE.113.000841 PMC4080303

[43] M. M. Seldin, S. Koplev, P. Rajbhandari, L. Vergnes, G. M. Rosenberg, Y. Meng, C. Pan, T. M. N. Phuong, R. Gharakhanian, N. Che, S. Mäkinen, D. M. Shih, M. Civelek, B. W. Parks, E. D. Kim, F. Norheim, K. C. Krishnan, Y. Hasin-Brumshtein, M. Mehrabian, M. Laakso, C. A. Drevon, H. A. Koistinen, P. Tontonoz, K. Reue, R. M. Cantor, J. L. M. Björkegren, A. J. Lusis, A strategy for discovery of endocrine interactions with application to whole-body metabolism. Cell Metab. 27, 1138–1155.e6 (2018). 29719227 10.1016/j.cmet.2018.03.015 PMC5935137

[44] C. Lavebratt, E. Dungner, J. Hoffstedt, Polymorphism of the AHSG gene is associated with increased adipocyte β2-adrenoceptor function. J. Lipid Res. 46, 2278–2281 (2005). 16024912 10.1194/jlr.M500201-JLR200

[45] S. Dasgupta, S. Bhattacharya, A. Biswas, S. S. Majumdar, S. Mukhopadhyay, S. Ray, S. Bhattacharya, NF-κB mediates lipid-induced fetuin-A expression in hepatocytes that impairs adipocyte function effecting insulin resistance. Biochem. J. 429, 451–462 (2010). 20482516 10.1042/BJ20100330

[46] A. K. Madiraju, D. M. Erion, Y. Rahimi, X. M. Zhang, D. T. Braddock, R. A. Albright, B. J. Prigaro, J. L. Wood, S. Bhanot, M. J. MacDonald, M. J. Jurczak, J. P. Camporez, H.-Y. Lee, G. W. Cline, V. T. Samuel, R. G. Kibbey, G. I. Shulman, Metformin suppresses gluconeogenesis by inhibiting mitochondrial glycerophosphate dehydrogenase. Nature 510, 542–546 (2014). 24847880 10.1038/nature13270 PMC4074244

[47] Y. Xiao, G.-P. Cai, X. Feng, Y.-J. Li, W.-H. Guo, Q. Guo, Y. Huang, T. Su, C.-J. Li, X.-H. Luo, Y.-J. Zheng, M. Yang, Splicing factor YBX1 regulates bone marrow stromal cell fate during aging. EMBO J. 42, e111762 (2023). 36943004 10.15252/embj.2022111762 PMC10152142

[48] A. K. Jayavelu, T. M. Schnöder, F. Perner, C. Herzog, A. Meiler, G. Krishnamoorthy, N. Huber, J. Mohr, B. Edelmann-Stephan, R. Austin, S. Brandt, F. Palandri, N. Schröder, B. Isermann, F. Edlich, A. U. Sinha, M. Ungelenk, C. A. Hübner, R. Zeiser, S. Rahmig, C. Waskow, I. Coldham, T. Ernst, A. Hochhaus, S. Jilg, P. J. Jost, A. Mullally, L. Bullinger, P. R. Mertens, S. W. Lane, M. Mann, F. H. Heidel, Splicing factor YBX1 mediates persistence of JAK2-mutated neoplasms. Nature 588, 157–163 (2020). 33239784 10.1038/s41586-020-2968-3

[49] C. Li, R.-I. Lin, M.-C. Lai, P. Ouyang, W.-Y. Tarn, Nuclear Pnn/DRS protein binds to spliced mRNPs and participates in mRNA processing and export via interaction with RNPS1. Mol. Cell. Biol. 23, 7363–7376 (2003). 14517304 10.1128/MCB.23.20.7363-7376.2003 PMC230327

[50] L. Yue, R. Wan, S. Luan, W. Zeng, T. H. Cheung, Dek modulates global intron retention during muscle stem cells quiescence exit. Dev. Cell 53, 661–676.e6 (2020). 32502396 10.1016/j.devcel.2020.05.006

[51] B. Liu, Y. Sun, Y. Zhang, Y. Xing, J. Suo, DEK modulates both expression and alternative splicing of cancer-related genes. Oncol. Rep. 47, 111 (2022). 35475534 10.3892/or.2022.8322 PMC9073418

[52] J. Chen, Y. Liu, S. Xia, X. Ye, L. Chen, Annexin A2 (ANXA2) regulates the transcription and alternative splicing of inflammatory genes in renal tubular epithelial cells. BMC Genomics 23, 544 (2022). 35906541 10.1186/s12864-022-08748-6 PMC9336024

[53] R. Wu, S. Cao, F. Li, S. Feng, G. Shu, L. Wang, P. Gao, X. Zhu, C. Zhu, S. Wang, Q. Jiang, RNA-binding protein YBX1 promotes brown adipogenesis and thermogenesis via PINK1/PRKN-mediated mitophagy. FASEB J. 36, e22219 (2022). 35195911 10.1096/fj.202101810RR

[54] A. C. Carpentier, D. P. Blondin, F. Haman, D. Richard, Brown adipose tissue-A translational perspective. Endocr. Rev. 44, 143–192 (2023). 35640259 10.1210/endrev/bnac015 PMC9985413

[55] A. M. Cypess, B. Cannon, J. Nedergaard, L. Kazak, D. C. Chang, J. Krakoff, Y.-H. Tseng, C. Schéele, J. Boucher, N. Petrovic, D. P. Blondin, A. C. Carpentier, K. A. Virtanen, S. Kooijman, P. C. N. Rensen, C. Cero, S. Kajimura, Emerging debates and resolutions in brown adipose tissue research. Cell Metab. 37, 12–33 (2025). 39644896 10.1016/j.cmet.2024.11.002 PMC11710994

[56] P. Dornbos, P. Singh, D.-K. Jang, A. Mahajan, S. B. Biddinger, J. I. Rotter, M. I. McCarthy, J. Flannick, Evaluating human genetic support for hypothesized metabolic disease genes. Cell Metab. 34, 661–666 (2022). 35421386 10.1016/j.cmet.2022.03.011 PMC9166611

[57] H. B. Cutler, J. Stöckli, S. Madsen, S. W. C. Masson, O. K. Fuller, T. B. Shum, D. E. James, Synteny – A high throughput web tool to streamline causal gene prioritisation and provide insight into protein function. bioRxiv 643559 [Preprint] (2025). 10.1101/2025.03.16.643559. PMC12749926 41462053

[58] E. T. Chouchani, L. Kazak, M. P. Jedrychowski, G. Z. Lu, B. K. Erickson, J. Szpyt, K. A. Pierce, D. Laznik-Bogoslavski, R. Vetrivelan, C. B. Clish, A. J. Robinson, S. P. Gygi, B. M. Spiegelman, Mitochondrial ROS regulate thermogenic energy expenditure and sulfenylation of UCP1. Nature 532, 112–116 (2016). 27027295 10.1038/nature17399 PMC5549630

[59] C. M. C. Andrés, J. M. Pérez de la Lastra, C. Andrés Juan, F. J. Plou, E. Pérez-Lebeña, Superoxide anion chemistry—Its role at the core of the innate immunity. Int. J. Mol. Sci. 24, 1841 (2023). 36768162 10.3390/ijms24031841 PMC9916283

[60] Y. Li, S. Mouche, T. Sajic, C. Veyrat-Durebex, R. Supale, D. Pierroz, S. Ferrari, F. Negro, U. Hasler, E. Feraille, S. Moll, P. Meda, C. Deffert, X. Montet, K.-H. Krause, I. Szanto, Deficiency in the NADPH oxidase 4 predisposes towards diet-induced obesity. Int. J. Obes. 36, 1503–1513 (2012). 10.1038/ijo.2011.279 22430302

[61] A. Fedorenko, P. V. Lishko, Y. Kirichok, Mechanism of fatty-acid-dependent UCP1 uncoupling in brown fat mitochondria. Cell 151, 400–413 (2012). 23063128 10.1016/j.cell.2012.09.010 PMC3782081

[62] I. G. Shabalina, E. C. Backlund, J. Bar-Tana, B. Cannon, J. Nedergaard, Within brown-fat cells, UCP1-mediated fatty acid-induced uncoupling is independent of fatty acid metabolism. Biochim. Biophys. Acta 1777, 642–650 (2008). 18489899 10.1016/j.bbabio.2008.04.038

[63] R. Westerberg, J. E. Månsson, V. Golozoubova, I. G. Shabalina, E. C. Backlund, P. Tvrdik, K. Retterstøl, M. R. Capecchi, A. Jacobsson, ELOVL3 is an important component for early onset of lipid recruitment in brown adipose tissue. J. Biol. Chem. 281, 4958–4968 (2006). 16326704 10.1074/jbc.M511588200

[64] C. A. Engelhard, S. Khani, S. Derdak, M. Bilban, J. W. Kornfeld, Nanopore sequencing unveils the complexity of the cold-activated murine brown adipose tissue transcriptome. iScience 26, 107190 (2023). 37564700 10.1016/j.isci.2023.107190 PMC10410515

[65] S. Vernia, Y. J. Edwards, M. S. Han, J. Cavanagh-Kyros, T. Barrett, J. K. Kim, R. J. Davis, An alternative splicing program promotes adipose tissue thermogenesis. eLife 5, e17672 (2016). 27635635 10.7554/eLife.17672 PMC5026472

[66] Y. T. Naing, L. Sun, The role of splicing factors in adipogenesis and thermogenesis. Mol. Cells 46, 268–277 (2023). 37170770 10.14348/molcells.2023.2195 PMC10183792

[67] X. Zhu, B. Lan, X. Yi, C. He, L. Dang, X. Zhou, Y. Lu, Y. Sun, Z. Liu, X. Bai, K. Zhang, B. Li, M. J. Li, Y. Chen, L. Zhang, HRP2-DPF3a-BAF complex coordinates histone modification and chromatin remodeling to regulate myogenic gene transcription. Nucleic Acids Res. 48, 6563–6582 (2020). 32459350 10.1093/nar/gkaa441 PMC7337902

[68] C. T. Herz, O. C. Kulterer, M. Prager, C. Schmöltzer, F. B. Langer, G. Prager, R. Marculescu, A. Kautzky-Willer, M. Hacker, A. R. Haug, F. W. Kiefer, Active brown adipose tissue is associated with a healthier metabolic phenotype in obesity. Diabetes 71, 93–103 (2022). 10.2337/db21-0475 34957487

[69] G. I. Smith, B. Mittendorfer, S. Klein, Metabolically healthy obesity: Facts and fantasies. J. Clin. Invest. 129, 3978–3989 (2019). 31524630 10.1172/JCI129186 PMC6763224

[70] P. L. Valenzuela, P. Carrera-Bastos, A. Castillo-García, D. E. Lieberman, A. Santos-Lozano, A. Lucia, Obesity and the risk of cardiometabolic diseases. Nat. Rev. Cardiol. 20, 475–494 (2023). 36927772 10.1038/s41569-023-00847-5

[71] Y. Yilmaz, O. Yonal, R. Kurt, F. Ari, A. Y. Oral, C. A. Celikel, S. Korkmaz, E. Ulukaya, O. Ozdogan, N. Imeryuz, E. Avsar, C. Kalayci, Serum fetuin A/α2HS-glycoprotein levels in patients with non-alcoholic fatty liver disease: Relation with liver fibrosis. Ann. Clin. Biochem. 47, 549–553 (2010). 20926473 10.1258/acb.2010.010169

[72] A. M. Hennige, H. Staiger, C. Wicke, F. Machicao, A. Fritsche, H.-U. Häring, N. Stefan, Fetuin-A induces cytokine expression and suppresses adiponectin production. PLOS ONE 3, e1765 (2008). 18335040 10.1371/journal.pone.0001765 PMC2258416

[73] D. J. Harney, A. T. Hutchison, Z. Su, L. Hatchwell, L. K. Heilbronn, S. Hocking, D. E. James, M. Larance, Small-protein enrichment assay enables the rapid, unbiased analysis of over 100 low abundance factors from human plasma. Mol. Cell. Proteomics 18, 1899–1915 (2019). 31308252 10.1074/mcp.TIR119.001562 PMC6731089

[74] J. Rappsilber, M. Mann, Y. Ishihama, Protocol for micro-purification, enrichment, pre-fractionation and storage of peptides for proteomics using StageTips. Nat. Protoc. 2, 1896–1906 (2007). 17703201 10.1038/nprot.2007.261

[75] V. Demichev, C. B. Messner, S. I. Vernardis, K. S. Lilley, M. Ralser, DIA-NN: Neural networks and interference correction enable deep proteome coverage in high throughput. Nat. Methods 17, 41–44 (2020). 31768060 10.1038/s41592-019-0638-x PMC6949130

[76] S. Hänzelmann, R. Castelo, J. Guinney, GSVA: Gene set variation analysis for microarray and RNA-seq data. BMC Bioinformatics 14, 7 (2013). 23323831 10.1186/1471-2105-14-7 PMC3618321

[77] A. Perdikari, G. G. Leparc, M. Balaz, N. D. Pires, M. E. Lidell, W. Sun, F. Fernandez-Albert, S. Müller, N. Akchiche, H. Dong, L. Balazova, L. Opitz, E. Röder, H. Klein, P. Stefanicka, L. Varga, P. Nuutila, K. A. Virtanen, T. Niemi, M. Taittonen, G. Rudofsky, J. Ukropec, S. Enerbäck, E. Stupka, H. Neubauer, C. Wolfrum, BATLAS: Deconvoluting brown adipose tissue. Cell Rep. 25, 784–797.e4 (2018). 30332656 10.1016/j.celrep.2018.09.044

[78] G. Yu, L.-G. Wang, Y. Han, Q.-Y. He, clusterProfiler: An R package for comparing biological themes among gene clusters. OMICS 16, 284–287 (2012). 22455463 10.1089/omi.2011.0118 PMC3339379

[79] W. Chen, Z. Xu, W. You, Y. Zhou, L. Wang, Y. Huang, T. Shan, Cold exposure alters lipid metabolism of skeletal muscle through HIF-1α-induced mitophagy. BMC Biol. 21, 27 (2023). 36750818 10.1186/s12915-023-01514-4 PMC9906913

[80] Y. Perez-Riverol, J. Bai, C. Bandla, D. García-Seisdedos, S. Hewapathirana, S. Kamatchinathan, D. J. Kundu, A. Prakash, A. Frericks-Zipper, M. Eisenacher, M. Walzer, S. Wang, A. Brazma, J. A. Vizcaíno, The PRIDE database resources in 2022: A hub for mass spectrometry-based proteomics evidences. Nucleic Acids Res. 50, D543–D552 (2022). 34723319 10.1093/nar/gkab1038 PMC8728295

