# Supplementary material for: Cold exposure stimulates cross-tissue metabolic rewiring to fuel glucose-dependent thermogenesis in brown adipose tissue
Source: Sci Adv. 2025 Jun 11;11(24):eadt7369. doi: 10.1126/sciadv.adt7369 (PMC12154194; doi:10.1126/sciadv.adt7369)
Supplement: Supplementary file 1 — Legends for supplementary data S1 to S3 [file sciadv.adt7369_sm.pdf]

Science Advances
AAAS

Supplementary Materials for

Cold exposure stimulates cross-tissue metabolic rewiring to fuel glucose-dependent thermogenesis in brown adipose tissue

Harry B. Cutler *et al.*

Corresponding author: Søren Madsen, soren.madsen@sydney.edu.au; David E. James, david.james@sydney.edu.au

Sci. Adv. **11**, eadt7369 (2025)
DOI: 10.1126/sciadv.adt7369

The PDF file includes:

Legends for supplementary data S1 to S3

Other Supplementary Material for this manuscript includes the following:

Supplementary data S1 to S3

Data S1. (separate file)
PhysiologicalData.xlsx: Raw data for Figure 1.

Data S2. (separate file)
ThermokineScoring.csv: Ranked list of thermokines between cold adapted tissues.

Data S3. (separate file)
WebTool.zip: File containing R shiny app file and associated data that are required to run the interactive data viewer (hosted at: https://bigproteomics.shinyapps.io/ColdAdaptation/)
